# A structurally conserved motif in γ-herpesvirus uracil-DNA glycosylases elicits duplex nucleotide-flipping

**DOI:** 10.1093/nar/gky217

**Published:** 2018-03-27

**Authors:** Christopher Earl, Claire Bagnéris, Kara Zeman, Ambrose Cole, Tracey Barrett, Renos Savva

**Affiliations:** Department of Biological Sciences, Institute of Structural and Molecular Biology, Birkbeck College, University of London, Malet Street, London WC1E 7HX, UK

## Abstract

Efficient γ-herpesvirus lytic phase replication requires a virally encoded UNG-type uracil-DNA glycosylase as a structural element of the viral replisome. Uniquely, γ-herpesvirus UNGs carry a seven or eight residue insertion of variable sequence in the otherwise highly conserved minor-groove DNA binding loop. In Epstein–Barr Virus [HHV-4] UNG, this motif forms a disc-shaped loop structure of unclear significance. To ascertain the biological role of the loop insertion, we determined the crystal structure of Kaposi’s sarcoma-associated herpesvirus [HHV-8] UNG (kUNG) in its product complex with a uracil-containing dsDNA, as well as two structures of kUNG in its apo state. We find the disc-like conformation is conserved, but only when the kUNG DNA-binding cleft is occupied. Surprisingly, kUNG uses this structure to flip the orphaned partner base of the substrate deoxyuridine out of the DNA duplex while retaining canonical UNG deoxyuridine-flipping and catalysis. The orphan base is stably posed in the DNA major groove which, due to DNA backbone manipulation by kUNG, is more open than in other UNG–dsDNA structures. Mutagenesis suggests a model in which the kUNG loop is pinned outside the DNA-binding cleft until DNA docking promotes rigid structuring of the loop and duplex nucleotide flipping, a novel observation for UNGs.

## INTRODUCTION

Kaposi’s sarcoma-associated herpesvirus (KSHV), also classified as HHV-8, is a double-stranded DNA virus belonging to the γ-herpesvirus subfamily. KSHV is the aetiologic agent of all forms of Kaposi’s sarcoma (KS) and has been linked with the lymphoproliferative disorders primary effusion lymphoma and multicentric Castleman’s disease (1–3). Dual phases of either latent epigenetic maintenance of the viral genome, or lytic replicative expansion, characterise the cellular continuance of γ-herpesviruses. During the latent phase, viral DNA repair and replication are achieved by the recruitment of host cell factors. This is a necessity since gene expression in the latent phase is restricted to apparently only between four and six viral proteins; all proteins expressed in the latent phase are associated with recruitment of host machinery for viral DNA maintenance, prevention of apoptosis and/or reactivation of the lytic phase (4). In contrast, activation of lytic viral replication results in the coordinated expression of over 80 transcripts associated with the suppression of interferon production, viral replication and assembly, and egress from the host cell (5). At the lytic stage of infection γ-herpesviruses express their own DNA repair and replication proteins. These include a uracil-DNA N-glycosylase (UNG), which has a canonical role in DNA repair as well as virus-specific roles in DNA replication.

The canonical role of UNGs is to repair uracil lesions in genomic DNA formed from cytosine deamination or introduced via misincorporation of dUTP (6). While U-A base pairs arising from misincorporation of dUTP are not miscoding, they nevertheless have the potential to perturb or prevent precise associations of proteins and DNA. More problematic are U:G mismatches, caused by the deamination of cytosine bases to form uracil, which promote G→A transition mutations if not repaired before the next round of replication. UNGs therefore initiate the base excision repair pathway in response to uracil in any DNA context. UNGs are enzymes that detect uracil bases with exquisite selectivity and cleave the N-glycosidic bond between the uracil base and the deoxyribose moiety leaving an abasic site (also known as an apurinic/apyrimidinic site, or AP site). An AP-endonuclease then creates a DNA backbone nick upstream of the abasic site and the resulting single-stranded gap is repaired by the sequential action of DNA repair associated polymerases and DNA ligases (7–9).

Almost all organisms encode enzymes with uracil-DNA glycosylase activity which share a common fold and are divided into six families on the basis of substrate specificity. UNGs, comprising family 1 of the uracil-DNA glycosylase superfamily, are the most ubiquitous of these enzymes and are well studied in terms of their structure and function (10). The catalytic C-terminal domains of UNGs (∼210–230 residues) are highly conserved at both the sequence and structure level while the N-terminal extensions are diverse and can be involved in subcellular localisation and protein–protein interactions (11). Sequence alignments of the conserved catalytic domain reveal sequence identity of 57%, between KSHV UNG and the UNG of the closely related γ-herpesvirus Epstein–Barr virus (EBV) [HHV-4]. KSHV UNG shares sequence identities of 45, 41 and 42% with the UNGs of Human cytomegalovirus (HCMV) [HHV-5], herpes simplex virus type 1 (HSV-1) [HHV-1] and human, respectively. The only structure available prior to this study of a γ-herpesvirus UNG is that of EBV UNG in complex with Ugi, a DNA-mimetic UNG-inhibiting protein encoded by the *Bacillus* bacteriophage PBS-1, which was used to aid UNG crystallisation (12).

The question of why γ-herpesviruses such as KSHV and EBV encode their own UNG when they are capable of recruiting the host enzyme is in part addressed by the observation that, while EBV UNG is an active DNA repair enzyme, it also serves as a structural element in the viral DNA replication complex (13). Despite the high degree of conservation in UNG sequence, structure and catalytic features, several viral UNGs also have non-canonical functions in DNA replication. As well as EBV UNG, the UNGs of HCMV and Vaccinia virus are indispensable for effective viral DNA replication (13–15) and the UNGs of HSV-1 and HCMV are associated with epigenetic maintenance (16,17). The detailed molecular basis of herpesvirus UNG involvement in DNA replication is not well studied. Investigation of the roles of virus-specific sequence differences in non-canonical biological roles could explain exactly why entire virus families, such as the herpesviruses, encode UNGs and why their UNGS differ in important motifs both from the host enzyme, and from other members of those viral families. In this study, that question has been addressed via structural and mechanistic analysis of the UNG encoded by KSHV, kUNG.

Although little published research has focused on kUNG itself, the role of EBV UNG in viral DNA replication has been shown to include translocation of UNG to the nucleus and co-localisation with the viral DNA polymerase in replication compartments. Direct interactions with members of the EBV replicative complex including the viral DNA polymerase and the DNA polymerase processivity factor are mediated by residues in the conserved C-terminal region of EBV UNG including the DNA binding cleft and the surrounding region (13). In an EBV infected HEK293-derived cell line, viral replication in EBV UNG knockouts is defective. Viral replication is fully restored by co-transfection with a catalytically inactive mutant of EBV UNG (Q91L, D91N) yet it is only partially restored by co-transfection with an UNG leucine loop DNA binding motif mutant of EBV UNG (H213L) (13).

**Figure 1.**
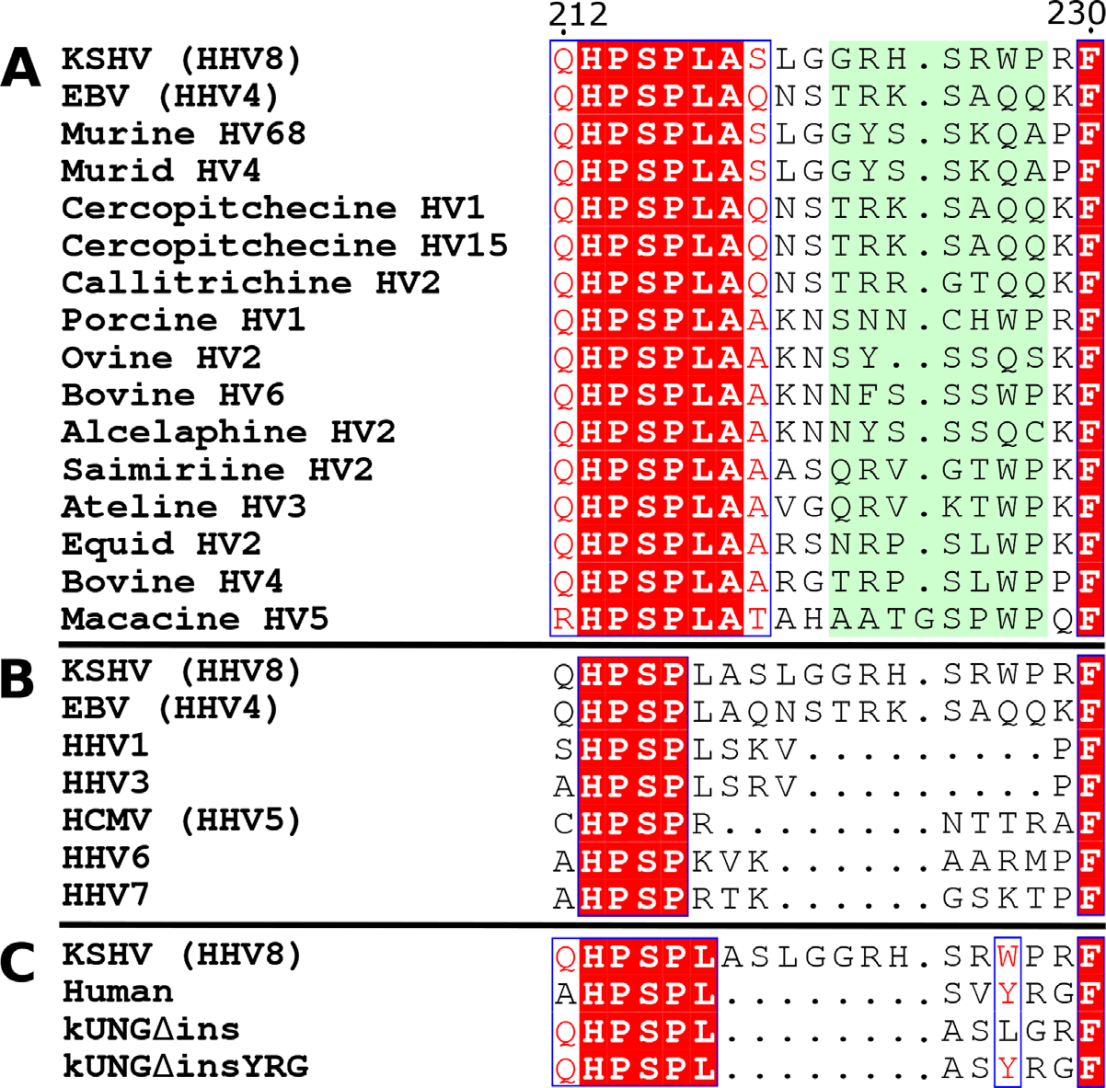
Sequence alignments of UNG leucine loops, adapted from ESPript web server outputs (19). Within each alignment, strictly conserved residues are written in white and highlighted in red. Residues with >70% conservation are surrounded by a blue box and written in red. (**A**) Leucine loop sequences of γ-herpesviruses with leucine loop extensions highlighted in pale green. (**B**) Leucine loop sequences of all known human herpesviruses. (**C**) Leucine loop sequences of kUNG, human UNG and kUNG mutants used in this study.

The UNG leucine loop is a conserved structural feature which is responsible for widening the DNA minor groove and flipping pyrimidines out of the base stack and, in the case of uracil, into the UNG active site for catalysis (18). Interestingly, γ-herpesvirus UNGs carry an extension to the C-terminal portion of the leucine loop. This embellishment of the leucine loop is present in all sequenced γ-herpesviruses and is absent in all other known UNGs. Although the length of the γ-herpesvirus leucine loop extension is well conserved at seven residues, Macacine HV5 being the only known exception with eight residues, there is poor sequence conservation in that motif (Figure 1).

The γ-herpesvirus UNG leucine loop extension is part of a key element of UNG DNA binding, and is implicated as a required component for viral replication. A structural investigation featuring a kUNG–dsDNA complex and kUNG in two alternative crystal packing arrangements of its unbound form was therefore used as a basis for studying the biological context of the γ-herpesvirus leucine loop. The crystal structures reveal that upon DNA binding, the loop extension favours the disc-like configuration previously observed in the EBV UNG–Ugi complex. The disc is stabilised by interactions involving residue 212 (almost exclusively a glutamine in γ-herpesvirus UNGs). Strikingly, however, two leucines within the motif flip both the uracil substrate and its orphaned partner out of the base stack. In order to perform this duplex nucleotide flipping, kUNG utilises a novel method of minor groove widening not seen in other UNGs. Duplex nucleotide flipping by an UNG is a novel observation: The orphan base is presented in a coordinated pose in the major groove of the DNA whilst concomitantly, the substrate uracil is flipped out of the base stack and into the kUNG active site for processing, as observed in all UNG–DNA structures. In this kUNG–dsDNA structure the major groove is more open than in previously reported UNG–dsDNA structures due to DNA backbone contacts, thereby improving potential accessibility of the displaced orphan base to other factors. To explore the key components of duplex nucleotide-flipping, a protein engineering approach was employed. Duplex nucleotide-flipping is not required for the canonical role of kUNG as a DNA repair enzyme, and the implication is that it serves another purpose in the lytic phase of the viral replicative cycle.

## MATERIALS AND METHODS

All enzymes and buffers for polymerase chain reaction (PCR), DNA modification and cloning were purchased from New England Biolabs (NEB). Synthetic oligonucleotides for PCR and crystallisation were supplied by Eurofins genomics, synthetic oligonucleotides for uracil-DNA glycosylase activity assays were purchased from Eurogentec. Unless stated otherwise, all other reagents were purchased from Sigma-Aldrich.

### Plasmid construction

A sequence encoding residues 19–257 of KSHV UNG (kUNGΔ18) was amplified by PCR from KSHV BC3 genomic DNA using Q5^®^ DNA polymerase with the forward and reverse primers GATATATGCTAGCGACGACCGGGATCTGTTAC and CATATATAAGCTTACTGCTCCAACAGGCCCC incorporating 5′ NheI and 3′ HindIII restriction sites, as underlined. The resulting fragment was digested with NheI-HF and HindIII-HF restriction enzymes. A pRSET-C plasmid (Life Technologies), previously modified to include an N-terminal 6×His tag and Tobacco Etch Virus (TEV) protease recognition site followed by an Nhe-I restriction site, was digested with the same restriction enzymes in the presence of CIP alkaline phosphatase. The KUNGΔ18 fragment was ligated into the cut vector using NEB Instant sticky end ligase master mix. The resulting plasmid, 6HKUΔ18, was propagated in NEB 5α cells and verified by fluorescent Sanger DNA sequencing (GATC Biotech). Mutants of 6HKUΔ18 were produced using the NEB Q5^®^ Site-Directed Mutagenesis Kit.

### Protein expression and purification

Recombinant expression from the 6HKUΔ18 wild-type and mutant plasmids was performed in NEB T7 Express LysY/I^q^ cells in LB broth (Miller) including 100 μg/ml ampicillin. Baffled 2.5 l Erlenmeyer flasks containing 800 ml of broth were inoculated 1/100 (v/v) from overnight cultures. Cultures were incubated at 37°C with shaking at 200 RPM until an absorbance at 600 nm of 0.3–0.4 was reached. The incubation temperature was reduced to either 18 or 30°C and, when the absorbance at 600 nm reached 0.8–1.0, overexpression was induced by the addition of 0.5 mM Isopropyl β-D-1-thiogalactopyranoside (IPTG). Growth was continued for 18–20 h at 18°C or 3 h at 30°C before cells were harvested by centrifugation. Cell paste was stored at −20°C for up to 1 month.

All purification steps were performed on ice with pre-chilled buffers unless stated otherwise. Frozen cell pellets were resuspended in lysis buffer containing 25 mM Tris–HCl, pH 7.5, 200 mM NaCl, 1 mM ethylenediaminetetraacetic acid (EDTA), 1% glycerol, 20 mM imidazole and one EDTA-free Roche complete protease inhibitor cocktail tablet per 50 ml. Cell suspensions were lysed by sonication. The lysate was centrifuged at 45 000 × *g* prior to loading onto a 1 ml HisTrap HP column (GE) which had previously been equilibrated with buffer **A** (25 mM Tris–HCl, pH 7.5, 200 mM NaCl, 1% glycerol, 20 mM imidazole). The HisTrap column was washed with 30 ml of buffer **A** including 50 mM imidazole prior to elution with 4 ml of buffer **A** supplemented with 500 mM imidazole. Tags were cleaved by the addition of ∼1/50 (wt/wt) TEV protease with overnight dialysis at 4°C against 4 l of buffer **B** (25 mM Tris–HCl, pH 7.5, 200 mM NaCl, 1 mM EDTA, 1% glycerol). The NaCl concentration of the dialysed sample was adjusted to 50 mM prior to loading onto a 1 ml HiTrap Heparin column (GE), previously equilibrated with 10 ml of buffer **C** (25 mM Tris–HCl, pH 7.5, 50 mM NaCl, 1 mM EDTA, 1% glycerol). The heparin column was washed with 10 ml of buffer **C** prior to elution with a 20 ml linear gradient of 50–1000 mM NaCl. Eluted fractions were analysed by Coomassie-stained sodium dodecyl sulphate-polyacrylamide gel electrophoresis and fractions judged to contain >95% pure KUNGΔ18 were pooled and concentrated using a Vivaspin-20 centrifugal concentrator (Sartorius) with a 10 kDa molecular weight cut-off. Soluble aggregates were removed by applying the sample to a Superdex 75 10/300 GL column (GE) in buffer **B**. Purified protein was concentrated to ∼1 mg/ml and stored at 4°C for up to 1 week, the overall yield of pure protein was ∼1 mg per liter of *Escherichia coli* culture. kUNG resulting from this purification including TEV protease digestion includes G19-Q255 of the native kUNG sequence preceded by a Ser-Ala-Ser (SAS) tripeptide which is an artefact from the expression plasmid.

### Oligonucleotides

Oligonucleotides used for crystallisation and assays are shown in Table 1. For crystallisation, single-stranded oligonucleotides were dissolved in water and stored at −80°C. Just prior to use, oligonucleotides were mixed in a 1:1 molar ratio in annealing buffer (20 mM Tris–HCl, pH 7.4, 100 mM NaCl, 20 mM MgCl_2_) and annealed by heating to 90°C, then cooling with a linear gradient to 10°C, over 160 min. Oligonucleotides (Eurofins Genomics) used for the ung activity assay, were prepared as U:G or U:A duplexes, by mixing the 5′-FAM-U and the 5′-G-BHQ1 or the 5′-A-BHQ1 oligonucleotides in a 1:1.1 molar ratio, and annealed as above before storage at −20°C.

**Table 1.** Synthetic oligonucleotides (in complementary pairs) used for crystallography and UNG activity assays

**5′FAM-idu_2** (crystallography)	FAM-5′-AAAGATAACATT-3′
	3′- TTCTA**U**TGTAA-5′
5′-FAM-U	FAM-5′- ATATATA**U**ATATATAACGCAGACACGTAGCAC-3′
5′-G-BHQ1 (U:G duplex for ung assay)	BHQ1–3′-TATATATATATGTATATATTGCGTCTGTGCATCGTG-5′
5′-FAM-U	FAM-5′- ATATATA**U**ATATATAACGCAGACACGTAGCAC-3′
5′-A-BHQ1 (U:A duplex for ung assay)	BHQ1–3′-TATATATATATATATATATTGCGTCTGTGCATCGTG-5′

FAM-5′ refers to the presence of a covalently linked 6-FAM label at the 5′ end of the oligonucleotide. BHQ1 refers to a covalently linked Black Hole Quencher 1 label at the 3′ end of the oligonucleotide. Uracil is shown in bold and underlined.

### Crystallisation and structure determination

For apo kUNG crystallisation, purified protein samples were concentrated to 10 mg/ml using an Amicon-ultra 3 kDa cut-off centrifugal concentrator (Merck Millipore) and used to set up 200 nl hanging drops containing a 1:1 ratio of protein to mother liquor using a number of commercially available screens (JCSG+, Index, PACT premier, Proplex, MIDAS, Morpheus). After incubation at 16°C for 5 days, diffracting crystals in two distinct forms were observed to grow in 0.2M lithium sulfate monohydrate, 0.1M Bis-Tris pH 6.5, 25% (w/v) PEG 3350 (space group C2) and 0.2M sodium chloride, 0.1M Bis-Tris pH 5.5, 25% (w/v) PEG 3350 (space group P2_1_). These were cryoprotected in mother liquor supplemented with 20% (v/v) ethylene glycol and flash-frozen in liquid nitrogen.

For kUNG–dsDNA complex crystallisation, purified protein samples were similarly concentrated to 8.5 mg/ml prior to addition of a 400 μM annealed 5′FAM-idu_2 stock (in DNA annealing buffer) to give an overall protein:DNA molar ratio of 1:1.2. After incubation for 60 min at room temperature, samples were further concentrated to 10 mg/ml with respect to protein and used to set up 3 μl hanging drops containing 1 μl of protein–DNA complex and 2 μl of mother liquor. After incubation at 16°C for 3–6 days, diffracting crystals grew from 100 mM sodium acetate, pH 4.5, 100 mM magnesium acetate, 8% (v/v) PEG 8000. These were cryoprotected in mother liquor supplemented with 20% (v/v) ethylene glycol and flash-frozen in liquid nitrogen.

Data sets were collected on beam line I04 at the Diamond light source using a PILATUS detector with an oscillation range of 0.10°, exposure time of 0.10 s and a transmission of 75% for 1800 images. Automated beam line processing was performed using XDS in the XIA2 pipeline (20,21). Further data processing was performed using XDS and DIALS, data were scaled and merged using AIMLESS (22,23).

The structures were initially phased by molecular replacement using the EBV UNG coordinates (PDB code: 2J8X, chain A) as a search model in Molrep using the Mr Bump pipeline (24,25). DNA was built manually using Coot. All structures were refined using Buster, Phenix and Refmac, see Table 2 for details (26–29).

**Table 2.** X-ray diffraction data collection and refinement statistics

	kUNG–dsDNA	kUNG crystal form 1	kUNG crystal form 2
PDB accession code	5NNU	5NN7	5NNH
Space group	P2_1_	P2_1_	C2
a, b, c, (Å)	82.08, 70.77, 140.19	42.87, 43.04, 57.39	105.20, 53.81, 40.81
α/β/γ (°)	90.00, 94.36, 90.00	90.00, 99.34, 90.00	90.00, 93.39, 90.00
Resolution range (Å)	49.20–2.97	56.63–2.50	52.51–2.20
Number of unique reflections	32502	7306	11642
*R*_merge_	0.144 (0.688)	0.06 (0.134)	0.089 (0.567)
Mean I/σ(I)	6.5 (1.9)	13.1 (6.2)	7.8 (2.1)
CC_1/2_	0.971 (0.656)	0.996 (0.978)	0.997 (0.858)
Completeness (%)	100.0 (100.0)	100.0 (99.9)	99.6 (99.7)
Multiplicity	3.4 (3.4)	3.3 (3.4)	3.1 (2.9)
**Refinement details**			
no. protein atoms	6973	3617	3365
no. DNA atoms	1760		
no. solvent atoms	111	57	85
*R*_work_/*R*_free_	0.239/0.264	0.207/0.252	0.199/0.244
Overall B factors (protein)	69.19	21.6	50.1
Overall B factors (DNA)	90.34		
Overall B factor (water)	51.17	16.9	46.9
**Ramachandran plot analysis**			
Favoured (%)	97.00	97.40	96.40
Allowed (%)	3.00	1.30	2.25
Outlier (%)	0.00	1.30	1.35
**Geometry analysis**			
RMSD bonds (Å)	0.015	0.006	0.0129
RMSD angles (°)	1.664	1.081	1.606

B factors are quoted per monomer: A, B, D, E for protein and S–Z for DNA chains.

### Uracil-DNA glycosylase activity assays

Uracil-DNA glycosylase activity was determined via a Förster resonance energy transfer (FRET)-based assay (30), in real time, with a BMG LABTECH FLUOstar OPTIMA plate reader using 485 nm excitation and 520 nm emission wavelengths. To achieve this, duplex DNA comprising a labelled 5′-fluorescent (FAM) uracil-containing strand and a 3′-quencher (BHQ1) partner strand (both adenine and guanine partner strands were utilised for each series) was employed. The FAM and BHQ1 oligonucleotides were formed into an offset duplex (see sub-section Oligonucleotides) and the corresponding U:G and U:A duplexes were mixed with kUNG and an excess of endonuclease IV (NEB), before recording changes in fluorescence due to the release of a short (low Tm) 5′-FAM ssDNA fragment upon cleavage of uracil by kUNG at 37°C. Accumulation of the liberated 5′-FAM ssDNA resulted in loss of quenching by BHQ-1 and an increased fluorescence signal due to FAM.

Optimum assay conditions were established via a series of tests performed using a range of substrate concentrations and varying wild-type kUNG concentrations. In order to obtain a linear increase of the initial velocity, wild-type kUNG at 6.65 nM concentration was found to be optimal under the assay conditions used.

Fluorescence intensity was measured using detection mode set at Plate Mode Kinetic. Gain was set at 5% using a well containing 400 nM 5′-FAM-U oligonucleotide. Quadruplicate (at a minimum) measurements were taken for each duplex (U:G or U:A) series. An increasing concentration of duplex DNA (26.6, 40, 53.3, 66.7, 100, 133.3, 200, 266.7 and 400 nM) in 50 μl buffer composed of 25 mM Tris, 100 mM NaCl pH 7.4, 0.1 mg/ml bovine serum albumin, 0.05% Tween-20, 20 mM MgCl_2_ was added to 96-well black *Microfluor 1* flat bottom microtiter plates (Thermo Scientific). Incubation of plates with gentle shaking for 15 min at 37°C was followed by addition of 100 μl of an enzyme mixture consisting of 6.65 nM of kUNG (WT or mutant) with an excess (1 μl) of Endonuclease IV (NEB). The plates were then loaded onto the plate reader before fluorescence data were collected at 30 s intervals over 35 min, at 37°C. In order to test if U:G and U:A duplexes possessed endogenous fluorescence, increasing concentrations of duplexes were set up as above with no protein present. Background fluorescence was observed at <20% of the signal observed for 5′-FAM-U alone: This signal did not increase with time and was subtracted from all measurements.

Maximum product produced (where fluorescence reached a plateau) was also recorded for all variant kUNG proteins using different substrate concentrations. Fluorescence increased linearly and calculated slopes were nearly identical when matching substrate was compared. In the cases of kUNGΔInsYRG with U:G and U:A duplexes, and Q212E with U:A substrate, maximum fluorescence was in the region of 25% of typical values; possible reasons for this are discussed in the results section. *V*_max_ is thus displayed as relative fluorescence units per sec (RFU s^−1^) rather than product molarity per sec (M s^−1^) to allow relative comparison. The kinetic calculation feature of MARS Data analysis software (BMG LABTECH) was used to determine the slope of the linear range between 4 and 8 min. These values (in RFU s^−1^) were then used in GraphPad Prism 7.02 for Windows (GraphPad Software, La Jolla, CA, USA, www.graphPad.com) to plot data against substrate concentration (nM) and then analyse the data using a nonlinear regression fit and Michaelis–Menten equation (Least squares fit).

## RESULTS

### The overall structure of the kUNG–dsDNA product complex

The structure of the kUNG–dsDNA complex was determined by molecular replacement using the coordinates of EBV UNG, derived from its complex with the UNG inhibitory protein Ugi (PDB code: 2J8X, chain A) (12). Electron density was absent or poor for the N-terminal residues encoded by the expression vector remaining after TEV protease treatment, as well as G19-V20 and the loop region between G157 and L165, which was resolved to varying degrees in each of the four copies present in the asymmetric unit (asymmetric unit shown in Supplementary Figure S1). Both of these unresolved regions are distal to the DNA-contacting surface of kUNG. Density for the DNA was clearly visible allowing all nucleotides to be manually built aside from the 5′ FAM-labelled dA. The deoxyribose moiety of the abasic site after uracil cleavage was modelled in the β-anomer conformation as seen in other UNG–dsDNA structures lacking a post-catalytic uracil in the active site (PDB codes: 2SSP (31) and 4UQM (32)). Of the four copies comprising the asymmetric unit, the complex comprising chains A, S and T was chosen for analysis since it has the lowest average B-factor and contains electron density for the greatest number of atoms in the leucine loop region.

The structure of kUNG in its apo form was solved in two different crystal forms. For the kUNG structure solved in space group C2, no electron density was observed for residues L217-S225 in the leucine loop. In the second kUNG structure, solved in space group P2_1_, residues R223-S225 in the leucine loop extension were disordered.

kUNG, in common with other known UNGs, comprises a single C-terminal globular domain made up of a four-stranded parallel β-sheet flanked by six α-helices, with an additional left-handed coil of three helices at the N-terminus. Structural alignments of residues 28–251 of kUNG to the equivalent residues of EBV UNG and hUNG yield global RMSDs with respect to Cα atoms of 0.92 Å and 0.55 Å, respectively. The number and position of N-terminal α-helices varies between different UNGs. In the case of EBV UNG there are two N-terminal helices, though for hUNG and kUNG there are three. The third helix in kUNG adopts a position similar to that of the second helix of EBV UNG but with Cα positions displaced by 1–3 Å when compared to the equivalent helix in hUNG (see Supplementary Figure S2 for alignments of kUNG with hUNG and EBV UNG). UNGs contain five conserved motifs required for efficient uracil-DNA glycosylase activity: the lytic water-activating loop, the proline-rich loop, the uracil binding motif, the glycine-serine motif and the leucine loop (33,34). The only significant structural variation in these conserved motifs, and in the whole C-terminal catalytic domain of kUNG, occurs in the leucine loop.

### kUNG elicits duplex nucleotide-flipping from the DNA double helix, exposing the orphan base to the exterior environment

The UNG leucine loop is named for the fact that it carries a strictly conserved leucine previously dubbed the ‘doorstop’ residue (35), whose sidechain is responsible for pushing the enzymatic substrate uracil base out of the DNA duplex and preventing return of the uracil to its usual position by steric hindrance. The base opposing the abasic site formed after uracil excision, dubbed the orphan base, is seen to remain in its usual position in the base stack in all other known structures with the exception of PDB code: 1EMH, the structure of the human UNG core catalytic domain (hUNG) in complex with an enzymatically resistant 2′-deoxy-pseudouridine-containing DNA (34). In 1EMH, the orphan base takes up an extrahelical position forming crystal contacts with symmetry related molecules. The orphan base conformation seen in 1EMH is therefore presumed to be a crystal artefact confirmed by the absence of this conformation in all other hUNG–dsDNA structures.

**Figure 2.**
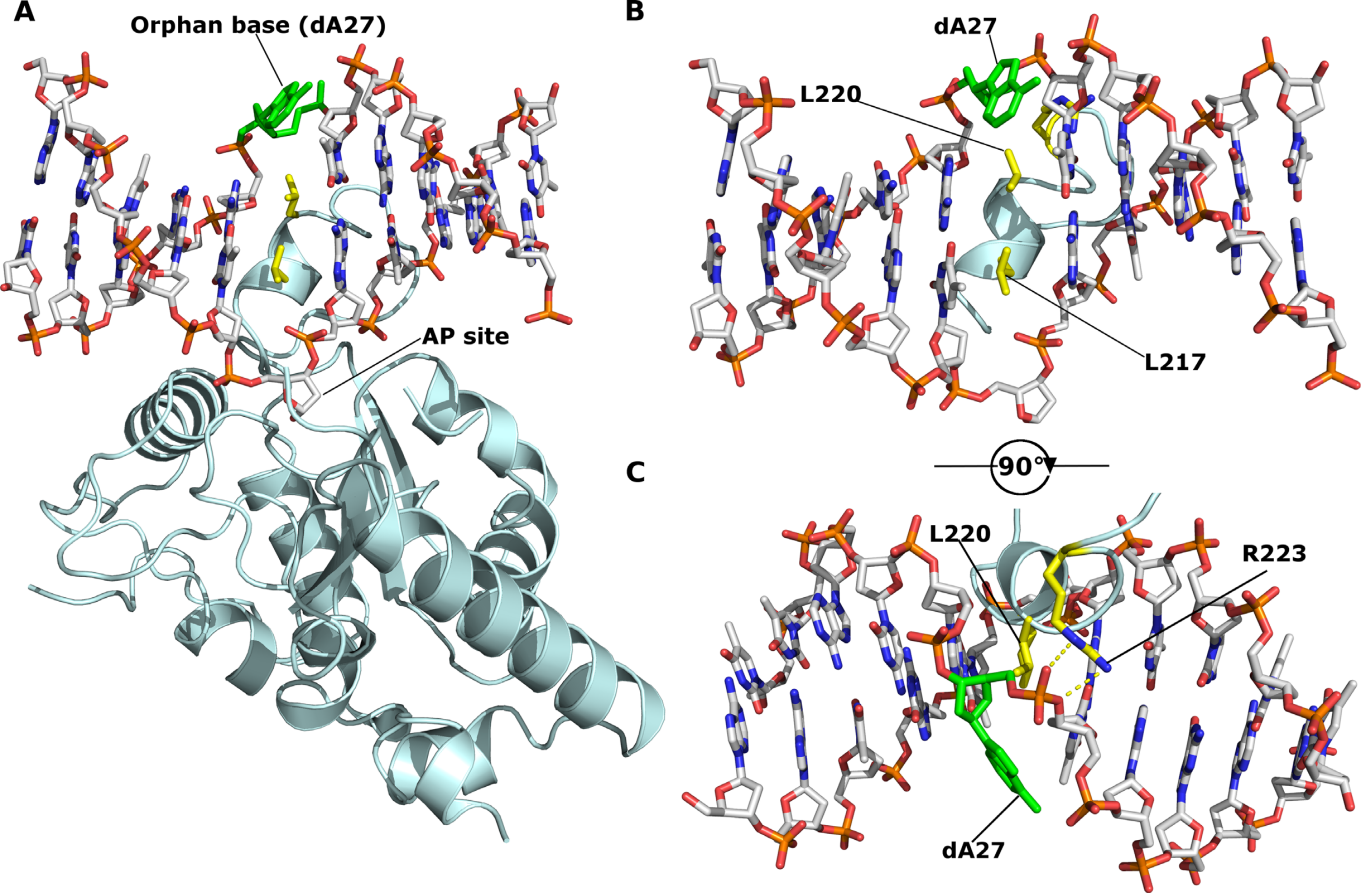
kUNG (pale cyan cartoon) in complex with DNA (sticks with grey carbons). Labelled residues, discussed below, are displayed as yellow sticks. The orphan base, dA27, is shown as green sticks. The AP site formed after uracil cleavage is labelled, no density was present for the cleaved uracil. (**A**) The overall architecture of the kUNG–dsDNA complex. (**B** and **C**) View of DNA and the kUNG leucine loop, only DNA and leucine loop residues are shown. Hydrogen bonds/electrostatic interactions are shown as yellow dashes. The leucine ‘loop’ includes a helical region that invades the minor groove presenting two leucine residues: L217, the canonical ‘doorstop’ residue which displaces the substrate uracil, and L220 which displaces the opposing ‘orphan’ base, dA27. The adenine base of dA27 is flipped out of the DNA duplex into the exterior environment, this is accompanied by the support of a strained backbone conformation by interactions with R223 of kUNG.

In the kUNG–dsDNA structure reported here, the orphan base of nucleotide dA27 is actively flipped out of the DNA duplex by the extended segment (common to all γ-herpesvirus UNGs) of the leucine loop, a feature not seen in any of the previously published structures of UNG–dsDNA complexes from human, *Deinococcus radiodurans, Thermus thermophilus* and Vaccinia virus (Figure 2). The extrahelical conformation of nucleotide dA27 is conserved in all four monomers of the crystal asymmetric unit where the base is solvent exposed and forms no contacts with neighboring molecules. Hydrogen bonds between the NH_2_ of the adenine base in dA27 and the phosphate of the neighboring nucleotide, dT26 rigidifies the orphan base in the crystal providing clear electron density (Supplementary Figure S3). This hydrogen bonding between the flipped-out adenine base with the phosphate of the neighboring nucleotide is similar to that observed for the flipped-out guanine observed in structures of endonuclease IV in complex with DNA, suggesting this to be an energetically favourable conformation for extrahelical purines (36) (Supplementary Figure S4).

Given the compressed nature of the DNA backbone in UNG–dsDNA complexes and the lack of base-pairing interactions available for orphan bases, they are liable to transition between extrahelical and intrahelical positions. Indeed, this fact is exploited in UNG activity assays which rely on the fluorescence increase upon liberation from the base stack of 2-aminopurine bases in the orphan position (37). In the kUNG–dsDNA structure however, both the adenine base and the deoxyribose moiety of dA27 are significantly displaced from the duplex stack with a distorted backbone conformation being stabilised by hydrogen bonding between the guanidinium head group of R223 in kUNG and the phosphate moiety of dA27 (Figure 2B and C, see Supplementary Table S1 for a summary of protein–DNA contacts). The position in the base stack usually occupied by dA27 is taken up by L220 of kUNG in a manner strikingly similar to that of the strictly conserved ‘doorstop’ leucine L217 which displaces the substrate uracil base from the DNA helix flipping it into the UNG active site.

### Relative kUNG activity for wild-type and engineered variants on duplex DNA substrates

Table 3 shows enzymatic data obtained from assays of kUNG variants investigated in this study. It is notable that mutations intended to weaken the function of the leucine loop extension via its structural destabilisation cause problems for catalytic function only with U:A substrates, whereas deletion of the loop extension reduces affinity and activity with both U:G and U:A substrates. The implications are discussed in the results and discussion, with respect to the rationale for mutagenesis versus a proposed model of functional significance of the leucine loop extension in γ-herpesviruses.

**Table 3.** Enzymatic parameters of wild-type and mutant kUNG using U:G and U:A duplexes

Enzymatic Parameters^a^	WT	Δ insYRG	Q212E	R229A	R223Q	R223S
U:G duplex						
*V*_max_ (RFU s^−1^)	0.857 ± 0.021	0.580 ± 0.017	0.718 ± 0.024	0.820 ± 0.017	0.857 ± 0.018	0.866 ± 0.017
*K*_m_ (nM)	48.870 ± 4.056	101.100 ± 7.463	63.007 ± 6.463	45.240 ± 3.323	47.450 ± 3.486	47.510 ± 3.148
U:A duplex						
*V*_max_ (RFU s^−1^)	0.872 ± 0.020	0.396 ± 0.018	0.153 ± 0.005	0.954 ± 0.022	0.899 ± 0.029	0.851 ± 0.025
*K*_m_ (nM)	44.99 ± 3.609	270.6 ± 22.42	145.4 ± 10.64	86.24 ± 5.464	162.6 ± 11.25	140.9 ± 9.494

^a^ *V*_max_ and *K*_m_ was determined by FRET-based assay. Values (mean ± SE) are the result of between 4 and 12 measurements. Data was analysed using a non-linear regression fit and the Michaelis–Menten equation (least squares fit).

### Orphan nucleotide-flipping is not required for UNG catalytic activity

To confirm whether or not nucleotide-flipping of the orphan base position is required for catalytic activity, site-directed mutagenesis was performed. Inspection of the structure reveals that backbone atoms of L220 penetrate the duplex deeply enough to at least partially occupy the usual position of the orphan base, therefore sterically, no point mutation at the L220 position could produce the desired effect of manipulating nucleotide-flipping of that orphan base position alone. A protein engineering approach was therefore pursued to abrogate duplex nucleotide-flipping in the kUNG–dsDNA system.

A mutant, kUNGΔins, was produced that lacked the leucine loop extension (G222-P228) resulting in a kUNG mutant with a leucine loop of the same length as that of hUNG (Figure 1 C). kUNGΔins had no detectable activity on dsDNA. Thermal shift experiments show that kUNGΔins has a folded hydrophobic core with a melting temperature of 44.5°C (±0.5°C), versus 47°C (±0.5°C) for wild-type kUNG (Supplementary Figure S5). The lower melting temperature for kUNGΔins suggests that it is less thermally stable than the wild-type protein and high fluorescence detected at lower temperatures indicates that kUNGΔins may be partially unfolded which may account for its lack of activity. Alternatively, kUNGΔins may be inactive due to the lack of ‘minor groove reading head’ residues seen in other UNGs, as explained below.

**Figure 3.**
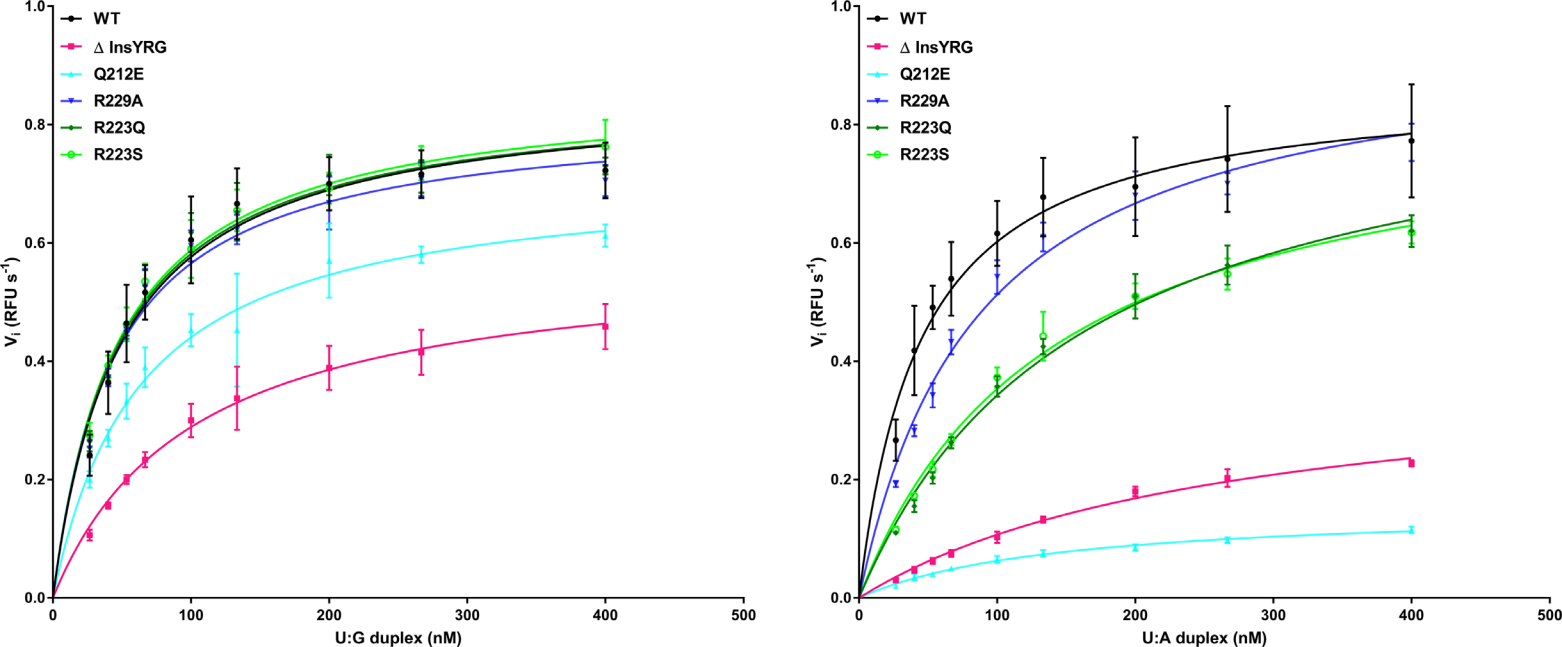
Relative enzymatic characteristics of wild-type kUNG, and mutants prepared for this study; 6.65 nM kUNG variant per assay: wild-type (black line, solid circle points), ΔinsYRG (pink line, solid square points), Q212E (cyan line, triangle points), R223Q (dark green line, diamond points), R223S (bright green line, hollow circle points), R229A (dark blue line, inverted triangle points). The substrate concentration is along the *x*-axis, and RFU s^−1^ is along the *y*-axis. (**A**) Substrate duplex DNA contains a U:G mismatch. (**B**) Substrate duplex DNA contains a U:A pair. A minimum of four measurements per duplex DNA series were performed; see text for full details.

**Figure 4.**
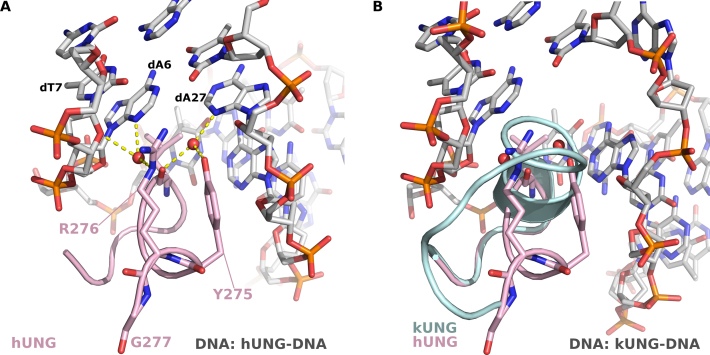
(**A**) Interactions between the hUNG ‘minor groove reading head’ and the DNA minor groove (PDB code: 1SSP). The hUNG leucine loop is shown as a pink cartoon with the Y275, R276 and G277 shown as sticks. DNA is shown in sticks with grey carbons, water molecules are shown as red spheres, hydrogen-bonds are shown as yellow dashes. R276 forms water-mediated hydrogen bonds with the adenine base of dA6 and the deoxyribose moiety of dT7, the nucleotide immediately 3′ of the uracil. Y275 forms a water-mediated hydrogen bond with the adenine base of dA27, the ‘orphan’ base. These interactions serve to widen the minor groove allowing the leucine loop to enter and perform nucleotide-flipping. (**B**) View [as in panel A] of the kUNG–dsDNA structure showing the kUNG leucine loop in pale cyan cartoon. The hUNG leucine loop is included [as in panel A]. The architecture of the kUNG leucine loop in this region is entirely different to that of hUNG with the kUNG protein backbone being inserted into the DNA duplex.

For the catalytic activity of UNGs, the leucine loop is required to widen the minor groove of the DNA via contacts from residues in the so-called ‘minor groove reading head’ (31). In hUNG, the minor groove reading head includes Y275 and the well-studied R276; the structure of the hUNG minor groove reading head is shown in Figure 4. Mutational analysis of hUNG R276 has shown this residue to be key to binding and activity on dsDNA and ssDNA (38,39) and an equivalent H275Y mutation in the UNG of Atlantic cod was severely deficient in activity on dsDNA (40). Considering those observations, and in light of our observations of kUNGΔins, we therefore engineered another mutant, kUNGΔinsYRG, which in addition to the leucine loop deletion, incorporated the aforementioned YR motif of the hUNG minor groove reading head. The kUNGΔinsYRG mutant is seen to partially restore UNG activity relative to the kUNGΔins mutant (Figure 3 and Table 3), but was poorly expressed and unstable with a limited half-life in cold storage. Enzymatic characteristics appear to indicate that kUNGΔinsYRG may undergo accelerated denaturation in standard assay conditions. Raw fluorescence measurements (data not shown) indicate that only ∼25% of the typical maximum fluorescence is observed with any duplex DNA substrate (versus wild-type, and all other mutants in this study, except Q212E with a U:A duplex DNA). Although the slope, when plotting fluorescence against substrate concentration (data not shown) remains comparable between measurements (this is the case with all active variants of kUNG in this study), there is substantial decay in fluorescent yield with kUNGΔinsYRG from the same preparation assayed from day to day; this decay effect is not seen with the wild-type or other [active] mutants studied. The implication is that the structural stability of the enzyme overall is perturbed by changes to the leucine loop region of UNG. These observations lend support to prior work (12,13,15), implicating that maintenance of the longer C-terminal insertion observed in γ-herpesviruses is at the potential cost of viral viability if its evolved character is disrupted.

### Exaggerated minor groove widening by kUNG

**Figure 5.**
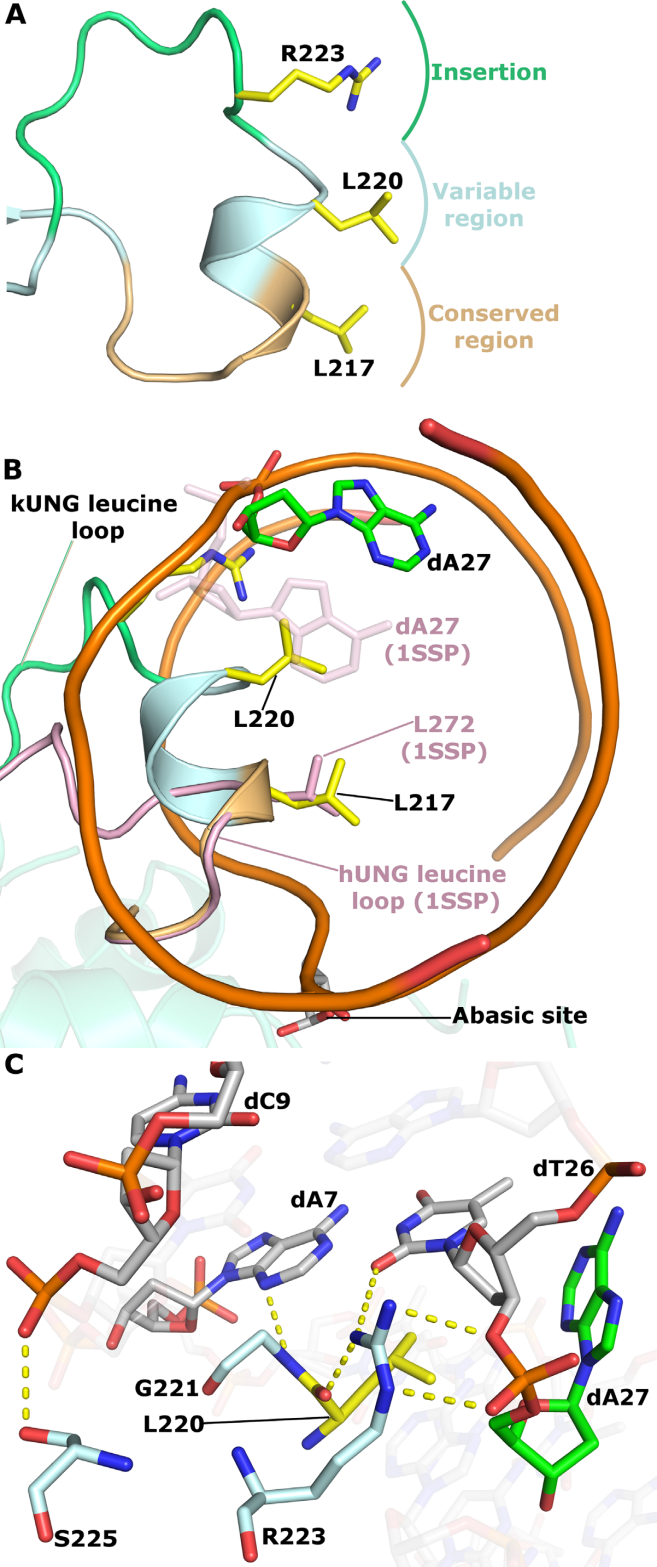
kUNG leucine loop structure. (**A**) Schematic of the conserved, variable and extension regions of the kUNG leucine loop. The strictly conserved ‘doorstop’ leucine L217, orphan nucleotide-flipping leucine L220 and key DNA-binding residue R223 sidechains are shown as sticks with yellow carbons. (**B**) View along the axis of the DNA double helix comparing kUNG and hUNG leucine loop positions, the DNA backbone is shown as an orange ribbon, most nucleotides are omitted for clarity. The kUNG leucine loop is coloured [as in panel A], the hUNG leucine loop, from PDB code: 1SSP (31), is shown in pink cartoon representation with the ‘doorstop’ leucine shown as pink sticks. The orphan dA27 nucleotide in the kUNG–dsDNA structure is shown in sticks with green carbons. The orphan base in 1SSP (faded pink sticks) takes its usual position in the base stack; this position would be precluded by steric hindrance from L220 of kUNG. (**C**) Stick representation of key protein–DNA interactions between the variable/extension regions of the leucine loop and the DNA minor groove. Protein residues are shown as sticks with pale cyan carbons, L220 is shown with yellow carbons. DNA is coloured [as in panel B]. Hydrogen bonds/electrostatic interactions are shown as yellow dashes. The guanidinium head group of R223 interacts with the phosphate group of dA27 and O2 of the dT26 base, as well as the carbonyl oxygen of L220. Further interactions from the kUNG leucine loop in the minor groove come from S225 and G221.

The wild-type kUNG leucine loop penetrates the DNA duplex more deeply than in all other published UNG–dsDNA structures (Figure 5). At the sequence level, the leucine loop is considered to consist of a conserved region (residues H213-L217 in kUNG), a variable region (residues A218-G221) and an extension region (residues G222-R229). Residues in the conserved region of the kUNG and hUNG leucine loops take up similar positions with Cα–Cα distances of less than 1 Å when kUNG and hUNG are aligned on the conserved catalytic core. Amino acid positions in the variable region progressively diverge with Cα–Cα distances having a range of 1.3–9.5 Å. The helical region of the leucine loop in kUNG is distinctly different being fully inserted into the duplex with both L220 and L217 occupying the usual positions of the U:A base pair (Figure 5).

The insertion of the leucine loop helical region for kUNG is permitted by the greater length of the loop and appears to be stabilised by extensive interactions between the guanidinium head group of R223 and dT26-dA27 in the DNA, as well as hydrogen bonding between the amide nitrogen of G221 and N3 of dA7 on the uracil-containing strand (Figure 5 and Supplementary Table S1). Modeling suggests the interaction between G221 and the base of dA7 is independent of the DNA sequence. R223 appears key to insertion of the leucine loop into DNA and facilitating the nucleotide-flipping of the orphan base position by stabilizing a twisted backbone conformation in this region. In light of this, the activity of mutants at R223, initially R223A and subsequently R223Q, and R223S, were investigated. Inspection of the structure suggests that Nη1 of R223 closely contacts the carbonyl oxygen of L220, thus the absence of this interaction in the R223A mutant may destabilise the leucine loop resulting in protein misfolding/ aggregation, and indeed recombinant kUNG R223A was insoluble and therefore not suitable for analysis. R223Q and R223S mutants however, result in little discernible kinetic difference in our assays involving U:G duplexes when compared to wild-type kUNG, yet these mutants appear to reduce affinity (and by implication, prolong residence) on U:A containing duplexes (Table 3).

### Additional protein–DNA contacts in kUNG reduce DNA kinking and confer an accessible major groove

**Figure 6.**
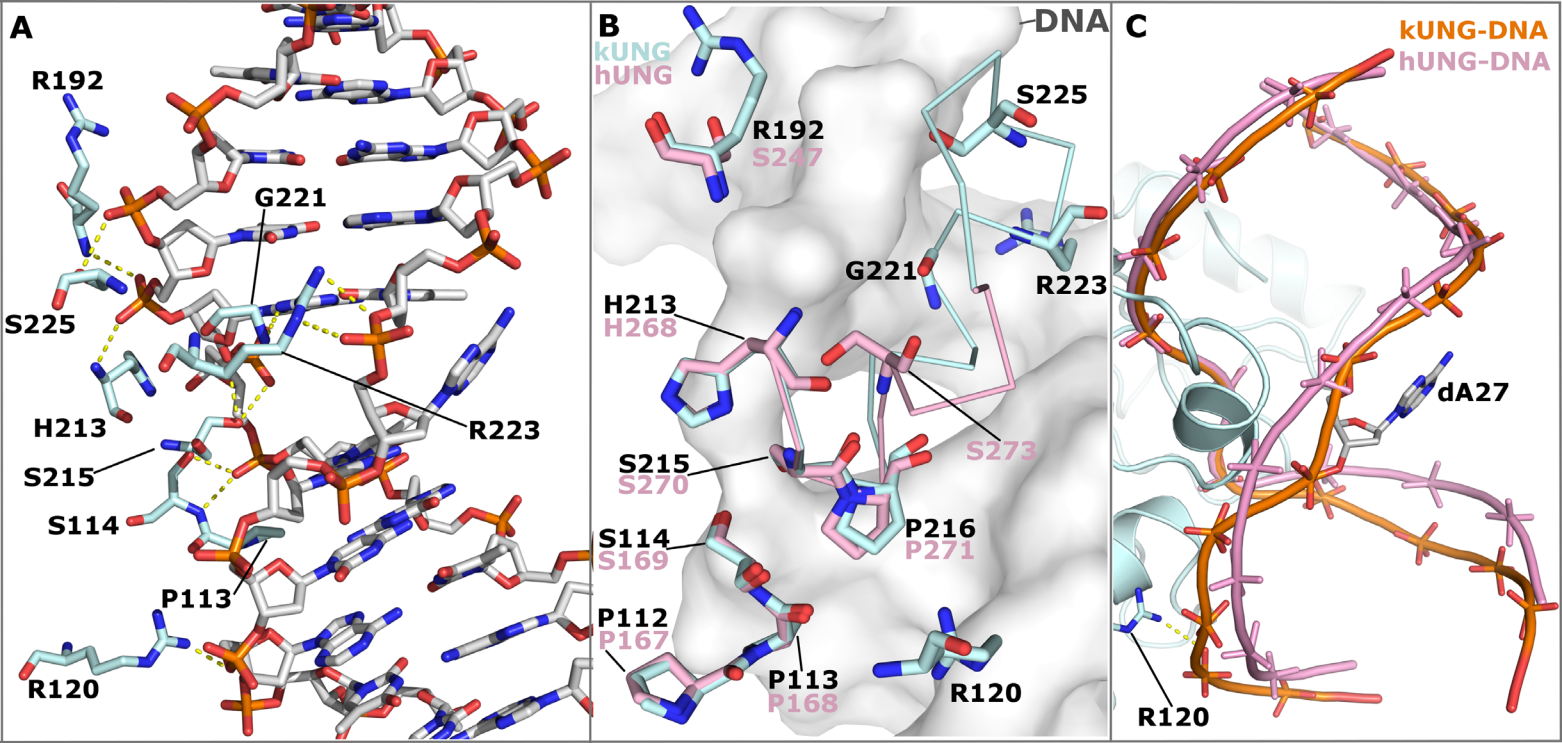
Protein–DNA contacts in the kUNG–dsDNA complex, see Supplementary Table S1 for details. (**A**) Stick representation showing DNA-contacting kUNG residues (pale cyan carbons), all other protein residues are omitted for clarity. Hydrogen bonds/electrostatic interactions are shown as yellow dashes. (**B**) Alignment of kUNG–dsDNA and hUNG–dsDNA (PDB code: 1SSP) complexes displaying structural conservation of the ‘Ser-Pro pinch' residues. kUNG residues are shown as sticks with pale cyan carbons, hUNG residues are shown as sticks with pink carbons. kUNG and hUNG leucine loop backbones are shown as ribbons in pale cyan and pink respectively. DNA from the kUNG–dsDNA complex is shown as a grey surface. Despite excellent overall conservation, the action of S273 in hUNG as a hydrogen bond donor is not mimicked by kUNG. Instead, contributions from G221, R223 and S225 provide ‘pinching’ interactions to compress the DNA backbone. R223 of kUNG provides an additional contact with the DNA backbone not seen in hUNG. (**C**) Comparison of the global DNA backbone conformation in enzyme–product complexes of kUNG (orange DNA) and hUNG (pink DNA). DNA backbones are shown as ribbon traces between phosphates. 3′ of the uracil (at the top of the image), the DNA backbone position is largely similar. 5′ of the uracil however, the DNA takes up a position closer to kUNG than in hUNG, a position supported by contact between R223 and the phosphate of dA29. There is a less pronounced kink in the DNA by kUNG than is seen in hUNG–dsDNA structures. Consequently, the flipped out orphan base dA27 is presented to the solvent in a more accessible major groove.

The exaggerated minor groove widening and duplex nucleotide-flipping observed in the kUNG–dsDNA structure is accompanied by additional protein–DNA contacts distal to the active site, and reduced global kinking of the DNA helix (Figure 6). The ‘Ser-Pro pinch’ mechanism observed in other structures consists of compression of the DNA backbone either side of the uracil via the concerted action of four serine, one glycine, and four proline residues (31). These canonical ‘pinch’ residues are structurally conserved between the kUNG and hUNG–dsDNA complexes with the exception of S273 in hUNG, located in the leucine loop variable region (Figure 6B and Supplementary Table S1). kUNG utilises several residues in the variable and extension regions of the leucine loop to produce a very similar DNA backbone conformation at the uracil position to that produced by hUNG (Supplementary Figure S6). In addition to contributions from R223 and G221, discussed in the previous section, the side-chain of S225 contacts the backbone in a manner which results in the DNA 3′ of the uracil being in a similar position to that observed in other structures despite this region being proximal to the γ-herpesvirus leucine loop extension. DNA in the region 5′ of the uracil takes up a position closer to the protein than in other structures, this results in kinking of the DNA being less pronounced than that observed in hUNG (Figure 6C). This straighter DNA conformation is likely stabilised by hydrogen bonding between the guanidinium head group of R120 and the DNA backbone. The reduction in DNA kinking by kUNG confers a less obstructed major groove than that found in other structures resulting in a precisely posed presentation of the flipped-out orphan base within an externally accessible cleft. This spatial arrangement contrasts with that observed in an endonuclease IV enzyme–product complex, in which the stably posed flipped-out orphan nucleotide is located in a highly constricted major groove (36) (Supplementary Figure S4). Orphan nucleotide-flipping by endonuclease IV therefore likely serves a purely catalytic role, to allow local backbone compression, which in turn permits the proper orientation of the opposing abasic site in the enzyme active site: the implication with kUNG, in contrast, is that orphan nucleotide flipping may serve a non-catalytic role.

### Conservation of γ-herpesvirus leucine loop extension structure upon occupation of the DNA-binding cleft

**Figure 7.**
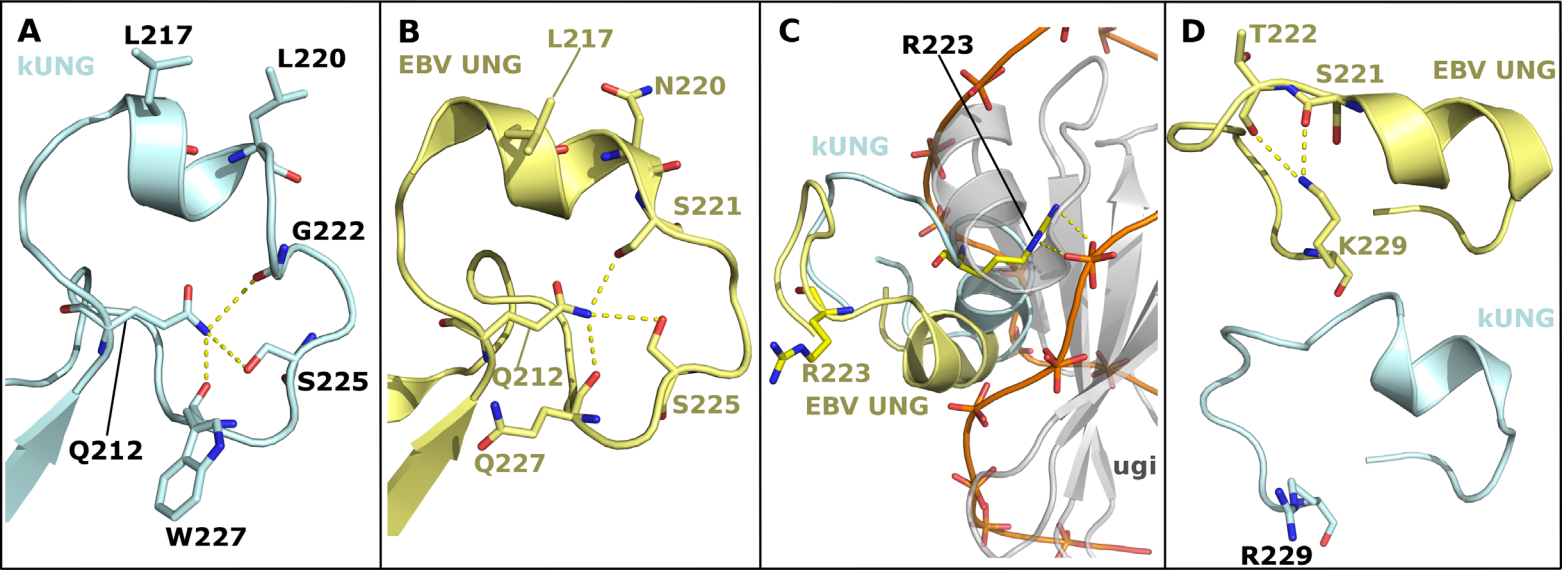
Comparison of leucine loop structures in the kUNG–dsDNA and EBV UNG–Ugi complexes (PDB code: 2J8X). (**A** and **B**) The leucine loops of kUNG (A, pale cyan) and EBV UNG (B, yellow) have similar structures including a rigid conformation of the extension region being centred on hydrogen-bonding with Q212. Residues involved in this hydrogen-bonding network and nucleotide-flipping residues are shown as sticks. Hydrogen bonds/electrostatic interactions are shown as yellow dashes. (**C**) Relative positions of the kUNG and EBV UNG leucine loops with the two UNGs aligned on conserved catalytic core residues. The kUNG leucine loop (pale cyan) invades the minor groove of the DNA (shown as an orange trace between phosphates). The EBV UNG leucine loop (yellow) is precluded from the same position as the kUNG loop by the bulky nature of Ugi (grey cartoon). R223 forms protein–DNA contacts in kUNG but protrudes into the solvent in the Ugi-bound EBV UNG structure. (**D**) Relative positions of R/K229. In the EBV UNG–Ugi structure (yellow), the side-chain of K229 is oriented towards the centre of the loop and interacts with T222 and S221. The side-chain of R229 of kUNG (pale cyan) forms no contacts with other atoms. R/K229 may act as a pre-catalytic ‘pinning’ residue to hold the loop away from the DNA binding cleft to allow DNA binding.

In the kUNG–dsDNA structure, the kUNG leucine loop extension (residues G222-R229) is ordered via a hydrogen bonding network centred on the side-chain of Q212 (Figure 7A). The architecture of the kUNG leucine loop extension closely resembles that seen for EBV UNG (PDB code: 2J8X chain A, Figure 7B). This is notable since γ-herpesvirus leucine loop extensions are not well conserved at the sequence level (Figure 1). Furthermore, the leucine loop in 2J8X is displaced from the DNA binding cleft of EBV UNG by Ugi, a bacteriophage-encoded UNG-inhibiting DNA-mimetic protein (Figure 7C). The Ugi protein was included in the study leading to 2J8X in order to aid crystallisation of EBV UNG (12). UNGs are inhibited by Ugi as a result of its occupation of the DNA binding cleft and sequestration of the ‘doorstop’ leucine sidechain in a hydrophobic pocket. Since Ugi is considerably bulkier than DNA, the EBV UNG leucine loop is precluded from taking up the position it does in the kUNG–dsDNA structure presented here. Conservation of the hydrogen bonding networks in the kUNG and EBV UNG leucine loop extensions suggests that the resulting structure of the extensions is rigid and may be conserved to aid catalytic activity by providing a stable framework to push the leucine loop into the DNA duplex when the DNA-binding cleft is occupied. A Q212E mutant protein was created to disrupt this framework, and in enzymatic assays, a relative decrease in affinity was observed, similar in magnitude to that observed for the R223(Q/S) mutants on U:A containing duplexes when compared to the wild-type protein. The implication is that in Q212E, other loop extension residues normally hydrogen bonded to Q212 are prevented from doing so. The loop extension would therefore be expected to take up alternative conformations in Q212E. However, similar to the R223(Q/S) mutants, Q212E displays little if any enzymatic difference to wild-type kUNG on U:G containing duplex substrates (Figure 3 and Table 3). Interestingly, Q212E also displays a low fluorescent yield at apparent plateau, around 33% relative to wild-type and other point mutants in this study, which is not dissimilar to that observed with kUNGΔinsYRG. However, notably the phenomenon is not linked to any apparent sample decay of Q212E, as is the case with kUNGΔinsYRG. Instead, it would appear that Q212E is simply a poor enzyme in the U:A containing duplex DNA substrate context. The implication is that when in contact with duplex DNA, differential hydrogen bonding with the disrupted loop extension side chains of Q212E may take place with flipped-out adenine, but not with guanine, presumably leading to increased residency on the substrate or more likely the product: Turnover would then be reduced.

The disc-shaped structure of the leucine loop extension centred on the side chain of Q212 was not observed in the two unbound kUNG structures. In the kUNG structure solved in space group C2, density was absent for the majority of the leucine loop (L217-S225). The leucine loop residues which were observed in the density adopted similar positions to those seen in the kUNG–dsDNA complex. For the second apo form of kUNG solved in space group P2_1_, the loop was partially ordered but had significant crystal contacts with symmetry-related molecules. In this structure, the side chain of L217 contacts a hydrophobic region near the N-terminus of a symmetry-related molecule and the leucine loop extension is precluded from forming the disc-like configuration seen in the kUNG–dsDNA structure by steric hindrance from the same symmetry-related molecule (Supplementary Figure S7). Taken together, the unbound kUNG structures suggest that the leucine loop extension is highly flexible in solution.

### Pre-catalytic leucine loop pinning by R229

Although sequence conservation amongst γ-herpesvirus UNG leucine loop extensions is low, R/K229 is somewhat conserved (Figure 1). In the kUNG–dsDNA structure the equivalent R229 side-chain takes up a solvent-exposed position forming no contacts with other atoms (Figure 7D and Supplementary Figure S8). In EBV UNG however, the equivalent K229 sidechain forms hydrogen bonds with the loop extension residues S221 and T222 effectively pinning the leucine loop away from the DNA binding cleft. Although EBV UNG–Ugi is a surrogate complex, the leucine loop in this structure can be envisaged to occupy a pseudo-pre-catalytic conformation and that R/K229 is present to pin the relatively bulky leucine loop of γ-herpesvirus UNGs away from the DNA binding cleft to permit pre-catalytic DNA binding. Consistent with this, a kUNG R229A mutant exhibits a modest decrease in substrate affinity when compared to the wild-type protein on U:A containing DNA but like the mutants, Q212E and R223(Q/S), there is negligible difference on U:G containing substrates (Table 3). This is a curious observation given that the solvent-exposed R229 sidechain appears to have no catalytic role when considering the wild-type kUNG–dsDNA structure alone (Supplementary Figure S8).

## DISCUSSION

An unexpected composite mechanism in a γ-herpesvirus uracil-DNA glycosylase has been uncovered that is associated with, yet distinct from, catalysis: Canonical nucleotide-flipping of a substrate deoxyuridine concomitant with nucleotide-flipping of its orphaned partner and coordinated presentation to the solvent environment of that orphan base. This is elicited by the leucine loop C-terminal extension found only in γ-herpesvirus UNGs. Notwithstanding sequence plasticity, the extended motif would appear to be a structurally conserved disc-shaped element in a substrate-docked UNG.

Previous structures show minimal infiltration of four or so leucine loop residues into the DNA duplex for the purpose of opening the minor groove, flipping the substrate deoxyuridine out of the duplex and preventing its return via steric hindrance from the ‘doorstop’ leucine sidechain. In contrast, nine residues of kUNG (S215-R223), including the entirety of a five-residue helix (P216-L220), intercalate between the DNA bases presenting two leucine sidechains to flip out both the substrate deoxyuridine and its partner nucleotide in a duplex nucleotide-flipping mechanism. The product is a precisely posed orphan base readily accessible to the exterior environment. Deletion of the leucine loop extension in kUNG necessitated the addition of the ‘minor groove reading head’ YR motif seen in hUNG, in order that the modified kUNG could retain some ability to widen the minor groove and insert the leucine loop into the DNA. The mutant, kUNGΔinsYRG, modelled with the intention of forming a canonical single nucleotide-flipping version of kUNG, has UNG activity but is structurally unstable with a short half-life. This suggests that the structural embellishment of the leucine loop required for duplex nucleotide-flipping is not a simple insertion to kUNG, and is required for its structural and functional integrity. Notably, the wild-type kUNG shows no apparent enzymatic preference for U:G or U:A duplex DNA substrates, which is as discovered previously with EBV UNG: The WT enzyme in that study behaved similarly for UA and UG (Km: 0.258/0.295, Kcat: 0.125/0.124) and moreover it had a higher affinity and turnover rate for ssDNA (Km 0.065, Kcat 0.275) (15). What is clear for kUNG is that enzymatic efficiency on duplex substrates is decreased in all cases of the mutants in which there is disruption or removal of the leucine loop extension (Table 3).

Duplex nucleotide-flipping is orchestrated by several motifs which work in concert to retain UNG enzymatic activity while presenting the orphan base to the solvent environment of the major groove in a rigid pose. The global DNA conformation and local configuration of the non-uracil-containing strand are markedly different in the kUNG–dsDNA structure when compared to hUNG–dsDNA complexes. The DNA backbone near the orphan base is twisted and kinked significantly to permit flipping of the base where the minor groove is opened more widely to accommodate the leucine loop, a conformation stabilised by the guanidinium head group of R223. An interaction between R223 and the DNA backbone sees the global DNA conformation in the kUNG–dsDNA structure less kinked than in previous analogous structures. Consequently, the major groove, where the orphan base is presented, is more open resulting in increased accessibility of this base to the exterior environment.

For UNG catalytic activity, the ‘Ser-Pro pinch’ method of backbone compression seen in other UNGs is retained almost residue by residue, thus catalysis remains conserved, albeit via a partially distinct mechanism. One serine residue in the leucine loop (S273 in hUNG) has no direct equivalent in kUNG but its effect is compensated for by other residues. Protein–DNA interactions involving G221, R223, and S225 of kUNG, as well as those conserved between kUNG and hUNG, result in the backbone conformation proximal to the substrate uracil produced by kUNG and hUNG being almost identical.

The deep infiltration of the kUNG leucine loop into DNA necessitates a different mechanism of DNA binding to that of other UNGs. Crystal structures and NMR analysis of hUNG/hUNG–dsDNA complexes reveal that hUNG switches from an open to a closed global conformation upon DNA binding which includes movement of the leucine loop approximately 3 Å in the same plane as the DNA bases (18,41). While this global closing mechanism may be present in kUNG, it is accompanied by a significant local swinging motion of the leucine loop about an axis orthogonal to that of the DNA double helix. In the EBV UNG–Ugi structure, the γ-herpesvirus leucine loop is displaced from the DNA binding cleft via interactions with residue K229, the equivalent of R229 in kUNG. A kUNG R229A mutant is observed to exhibit a modest decrease in substrate affinity compared to the wild-type enzyme on U:A but not U:G containing duplexes (Table 3). A possible interpretation is that R229 may dampen the mobility of the leucine loop to favour conformations away from the DNA binding cleft prior to catalysis and that upon DNA binding, the loop would swing into the DNA duplex. This proposed motion may be aided by the rigid nature of the leucine loop extension in a disc-like configuration owing to a hydrogen bonding network centred on Q212, as evidenced by a relative decrease in affinity for U:A containing, duplex DNA in the kUNG Q212E mutant. This is supported by conservation of the local conformation of the loop between the kUNG–dsDNA structure and the EBV UNG–Ugi structure despite the differing global conformations of the loop. However, the absence of electron density for the leucine loop insertion in the apo kUNG C2 crystal form, and partial ordering of the leucine loop along with significant crystal contacts in the P2_1_ crystal form indicate that the leucine loop is very likely highly mobile in solution. Interactions with the sidechain of Q212 include contacts with the carbonyl oxygen of two residues in kUNG and one residue in EBV UNG. The conserved length of the γ-herpesvirus UNG leucine loop (Figure 1), restricts leucine loop extension backbone atoms to positions close to the Q212 sidechain. As such, interactions from carbonyl oxygens are likely to be available to the Q212 sidechain with a high tolerance of sequence plasticity. This may explain why there is very low sequence conservation amongst γ-herpesvirus UNG leucine loop extensions while the local conformation of these extensions is structurally conserved between kUNG and EBV UNG. The leucine loop of Macacine herpesvirus 5 varies from all other γ-herpesvirus UNG leucine loops in two ways: the Macacine UNG leucine loop extension contains eight residues rather than seven, and the equivalent of Q212 in kUNG is an arginine residue in Macacine herpesvirus 5. That these two outliers existing in the same UNG lends weight to the model of Q212 in kUNG forming a frame which supports the leucine loop extension in a ring conformation surrounding the Q212 side chain in kUNG. In Macacine herpesvirus 5, the longer leucine loop extension could form a ring of a larger radius than that in kUNG which might thus accommodate a longer arginine side chain at its centre.

Presentation of the flipped orphan base requires infiltration of the leucine loop into the DNA duplex. For hUNG, this infiltration does not necessarily require the presence of a uracil in the DNA. Discrimination between uracil and thymine by UNG occurs in a pyrimidine ‘sieving pocket’ adjacent to the UNG active site. Thymine is precluded from entering the active site itself while uracil may enter for processing, producing an enzyme–product complex as in the kUNG–dsDNA structure (42). Importantly, the leucine loop in hUNG is fully inserted into the minor groove when a thymine occupies the pyrimidine sieving pocket. Furthermore, protein–DNA interactions from the leucine loop near the pyrimidine sieving pocket are all from residues in the conserved portion of the loop. The kUNG leucine loop is therefore suspected to be capable of infiltrating the duplex and flipping out both the interrogated, and the partner base, whether the partner base is a true orphan base, as in the kUNG–dsDNA structure, or the adenine of a T-A base pair. This may have relevance to the fact that an A-T rich palindromic sequence of 18 bp is suggested to serve as an origin for lytic phase replication in KSHV, and is the known binding site for other lytic phase replication factors such as K8 (43). Duplex nucleotide-flipping of a T-A base pair can therefore be readily envisaged from our knowledge of UNG scanning behaviour. Although we find no substrate preference for U:G or U:A in duplex DNA substrates for wild-type kUNG, all mutations affecting the structurally conserved leucine loop extension appear to have affinity effects on U:A containing duplex DNA substrates, but not to any great extent in a U:G context. The implication is that the leucine loop extension has evolved for a purpose other than catalysis *per se*, but the pressure to maintain it favours unbiased catalytic proficiency. The primary and secondary functions of γ-herpesvirus UNGs, namely scanning and catalytic proficiency, and duplex nucleotide flipping, are thus rendered mutually indispensable. For canonical UNGs, the relative activity and affinity for single-stranded substrates versus U:A containing substrates is unchanged in mutants of the ‘doorstop’ leucine (L191 in *E. coli* UNG, L217 in kUNG) (44). The role of the leucine loop for canonical UNGs is therefore proposed to be one of stabilizing an enzyme–substrate complex after the ‘Ser-Pro pinch’ mechanism has presented the substrate U in the active site (35,44,45). In contrast, kUNG appears to require its leucine loop to ‘push’ the substrate U and the orphaned partner base into the major groove side, as evidenced by the kinetic parameters for the Q212E/R229A/R223Q/R223S mutants being compromised for a substrate containing a U:A base pair, but not for a U:G wobble pair. The requirement in kUNG for the leucine loop to ‘push’ the substrate U out of the base stack is a distinct feature of kUNG compared to other UNGs, and this mechanism may exist in order to couple duplex nucleotide flipping to the catalytic activity of kUNG thereby mutually maintaining these two distinct functions of kUNG.

The adaptation of the leucine loop for orphan nucleotide-flipping suggests significant evolutionary pressure on γ-herpesvirus UNGs to precisely extrude the orphan base into the external environment. Along with the majority of structural features of the host cell UNG, kUNG retains canonical enzymatic activity and differences within the DNA-interacting motifs are compensated for elsewhere to produce the same DNA conformation around the substrate uracil. The retention of enzymatic features with addition of a structured motif to flip the orphan base out of the duplex into a rigid solvent-accessible pose in a widened major groove, are suggestive of a conserved biological function for the flipped orphan base. The viral DNA polymerase and its associated processivity factor have been shown to bind γ-herpesvirus UNGs close to their DNA binding motifs in a process essential for viral DNA replication (13). It may be conjectured that the DNA conformation observed in the kUNG–dsDNA structure could play a part in this process and may permit binding of an element of the viral replisome. In other words, a γ-herpesvirus UNG performing pyrimidine-scanning/sieving at the viral origin of replication could act as a sensor for replisome initiation. The insights provided by the kUNG–dsDNA structure and related studies would now benefit from *in vivo* dissection to ascertain the exact role of duplex nucleotide-flipping in the lytic phase of the viral lifecycle.

## DATA AVAILABILITY

Structures have been deposited in the PDB with accession codes: 5NNU (kUNG–dsDNA complex), 5NN7 (apo-kUNG in space group P2_1_) and 5NNH (apo-kUNG in space group C2), respectively. Supplementary Figure S9 comprises global validation metrics of the depositions, from validation reports provided by PDBe upon submission of the structures.

## Supplementary Material

Supplementary Data
