# Supplementary material for: A structurally conserved motif in γ-herpesvirus uracil-DNA glycosylases elicits duplex nucleotide-flipping
Source: Nucleic Acids Res. 2018 Mar 27;46(8):4286–300. doi: 10.1093/nar/gky217 (PMC5934625; doi:10.1093/nar/gky217)
Supplement: Supplementary Data [file gky217_supp.pdf]

Supplementary Information

Table S1:

Hydrogen bonding and electrostatic interactions between kUNG/hUNG and DNA. Conserved interactions are aligned where possible. Divergence in sequence and structure necessitates the separation of minor groove widening interactions. The lack of observed water-mediated interactions between kUNG and DNA is likely due to the comparatively lower resolution of the kUNG-dsDNA model.

**kUNG**					**hUNG**						
**Residue**	**Atom**	**Nucleotide**	**Atom**	**Distance (Å)**	**Residue**	**Atom**	**Nucleotide**	**Atom**	**Distance (Å)**		
S114	Oγ	AAB6	O1'	2.7	S169	Oγ/NH	AAB5	OP1	2.7/2.8		**Pinch**
R120	Nη2	dA29	03'	3.3	-	-	-	-	-		
R192	N	dT8	OP2	2.8	S247	NH	dT7	OP2	2.8		
H213	N	dT8	OP1	2.8	H268	NH	dT7	OP1	3.0		
S215	Oγ	dA7	OP2	3.0	S270	Oγ	dA6	OP1/O5'	3.1/3.1		
G221	N	dA7	N3	3.3	-	-	-	-	-		
A218	-	-	-	-	S273	Oγ	dA6	O3'/O5'	3.02/3.3		
G221	NH	dA7	N3	3.3	-	-	-	-	-		**kUNG minor groove widening**
R223	Nε	dA26	OP1	3.1	-	-	-	-	-		
R223	Nη2	dT25	03'	3.1	-	-	-	-	-		
R223	Nη1	dT25	O2	3.4	-	-	-	-	-		
S225	Oγ	dC9	OP1	3.0	-	-	-	-	-		
-	-	-	-	-	Y275	OH	dA27	N3	2.6/2.7	(water-mediated)	**hUNG minor groove widening**
-	-	-	-	-	R276	Nε	dA6	N3	2.7/2.9	(water-mediated)	
P216	-	-	-	-	P271	O	dA28	N3	2.9/3.0	(water-mediated)	
L217	-	-	-	-	L272	O	dA27	N3	2.6/2.7	(water-mediated)	

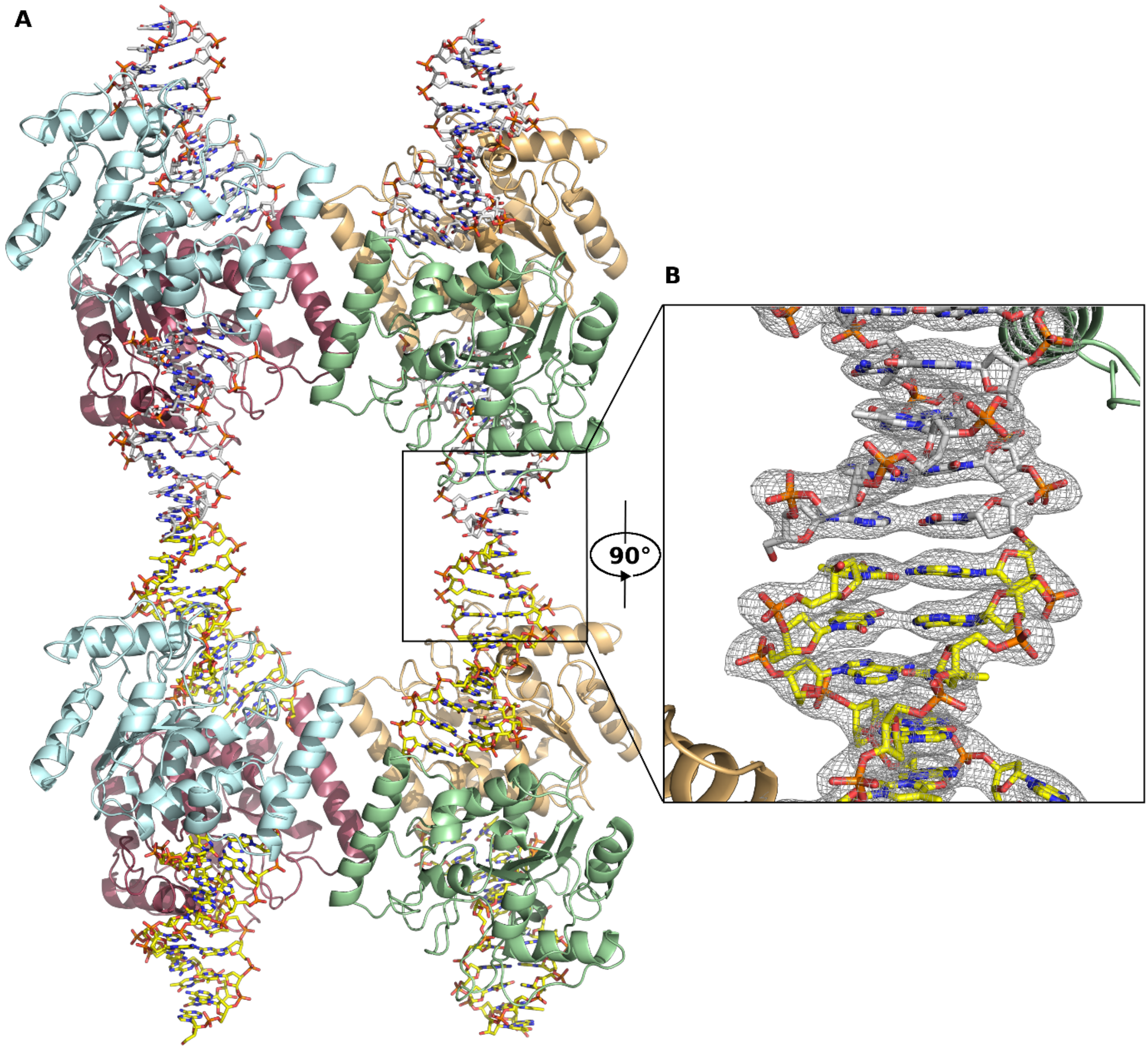

Figure S1:

Asymmetric unit and DNA base-stacking crystal contacts in kUNG. **A**: Two asymmetric units (ASUs) of kUNG displaying DNA-mediated crystal contacts. Each ASU consists of four copies of the kUNG-dsDNA complex with protein monomers coloured in pale cyan, maroon, green, and gold. DNA is shown as sticks with grey carbons in one copy of the ASU, and yellow carbons in the second copy. **B**: Base-stacking crystal contacts between the DNA of two asymmetric units. Electron density is shown as a grey mesh representation of a simulated annealing 2Fo-Fc omit map contoured at 1 σ with a carving distance of 1.6 Å.

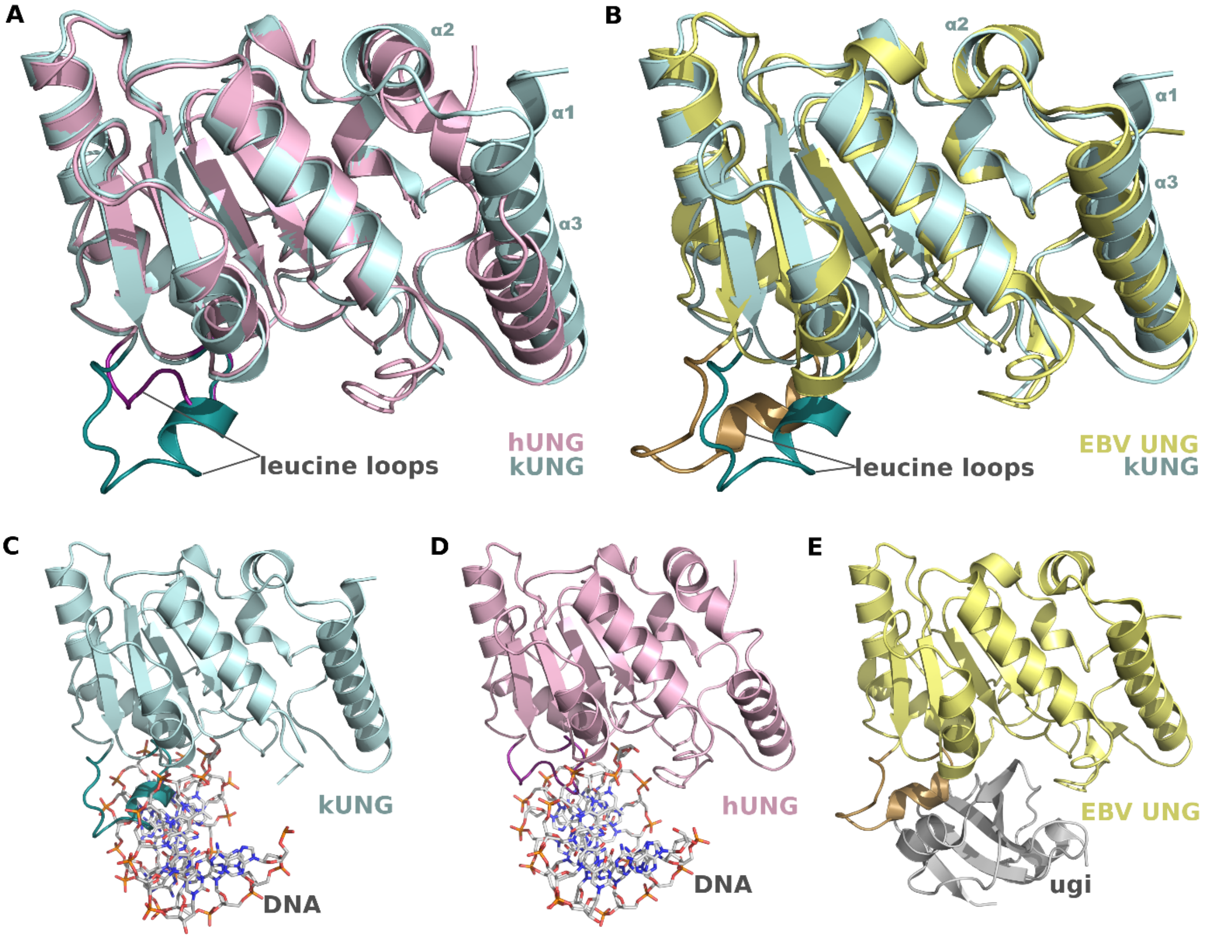

Figure S2:

A: kUNG (pale cyan cartoon) superposed with hUNG (pink cartoon, PDB entry: 1SSP). Leucine loops are coloured in dark cyan and dark pink respectively. **B**: kUNG, as in **A**, superposed with EBV UNG (yellow cartoon, PDB entry: 2J8X), the EBV UNG leucine loop is coloured dark gold. For clarity in A and B, DNA from the kUNG-dsDNA structure, DNA from 1SSP, and ugi from 2J8X have respectively been omitted. **C/D/E**: Individual cartoon views of the UNGs superposed in **A/B** including DNA (sticks) and ugi (yellow cartoon). The only major structural differences between UNGs are seen in the leucine loops and the angle of the 2nd/3rd N-terminal helices, a common structural variation in UNGs. Despite kUNG having a higher sequence identity with EBV UNG than hUNG, the overall structure of kUNG in the kUNG-dsDNA complex is more similar to 1SSP than 2J8X; structural alignments on Cα atoms yield global RMSDs of 0.55 Å between kUNG and 1SSP and 0.91 Å between kUNG and 2J8X. This is likely due to the kUNG-dsDNA structure and 1SSP both being enzyme-product complexes and therefore having a closed conformation as the protein ‘grips’ DNA.

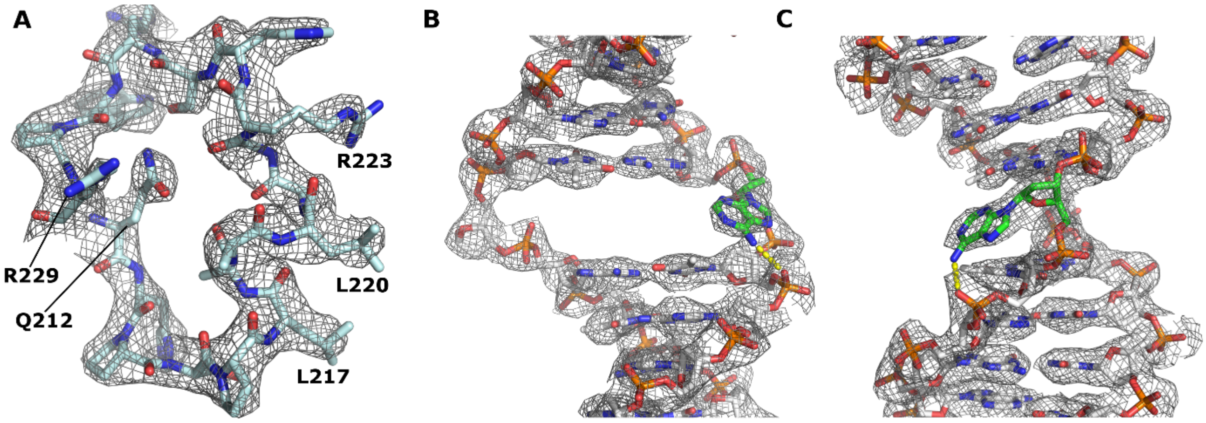

Figure S3:

Electron density for the kUNG leucine loop and DNA in the region of duplex nucleotide-flipping. Electron density is shown as a grey mesh representation of a simulated annealing 2Fo-Fc omit map contoured at 1 σ with a carving distance of 1.6 Å. Protein residues are shown as sticks with pale cyan carbons, DNA is shown as sticks with grey carbons in most nucleotides and green carbons in the case of the orphan nucleotide dA27. **A**: Electron density for the leucine loop, key residues discussed in the main text are labelled. **B/C**: Electron density for DNA. Hydrogen bonding between the orphan base of dA27 and the phosphate group of the neighbouring nucleotide (shown yellow dashes) stabilises the base in the crystal providing clear electron density.

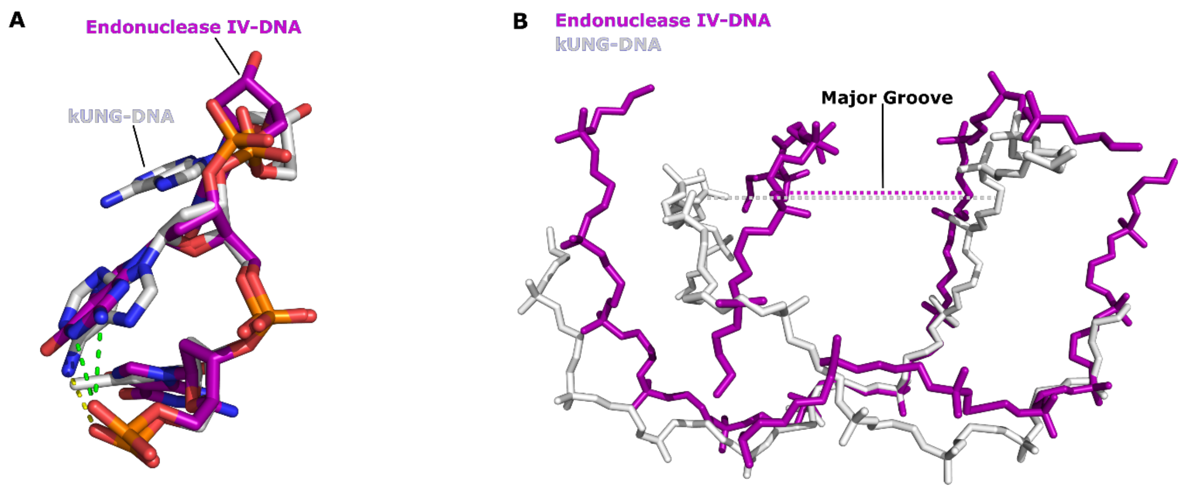

Figure S4:

Comparison of DNA backbone conformations in the kUNG-DNA (grey) and Endonuclease IV-DNA (magenta) product complex structures (Endonuclease IV-DNA complex from PDB entry 1QUM (34)). **A**: The extrahelical orphaned nucleotide takes up a similar position in the two structures, with hydrogen bonds formed with neighbouring phosphates (yellow dashes for kUNG-DNA, green dashes for endonuclease IV-DNA). **B**: The major groove in the kUNG-DNA structure is less constricted than that in the endonuclease IV-DNA complex.

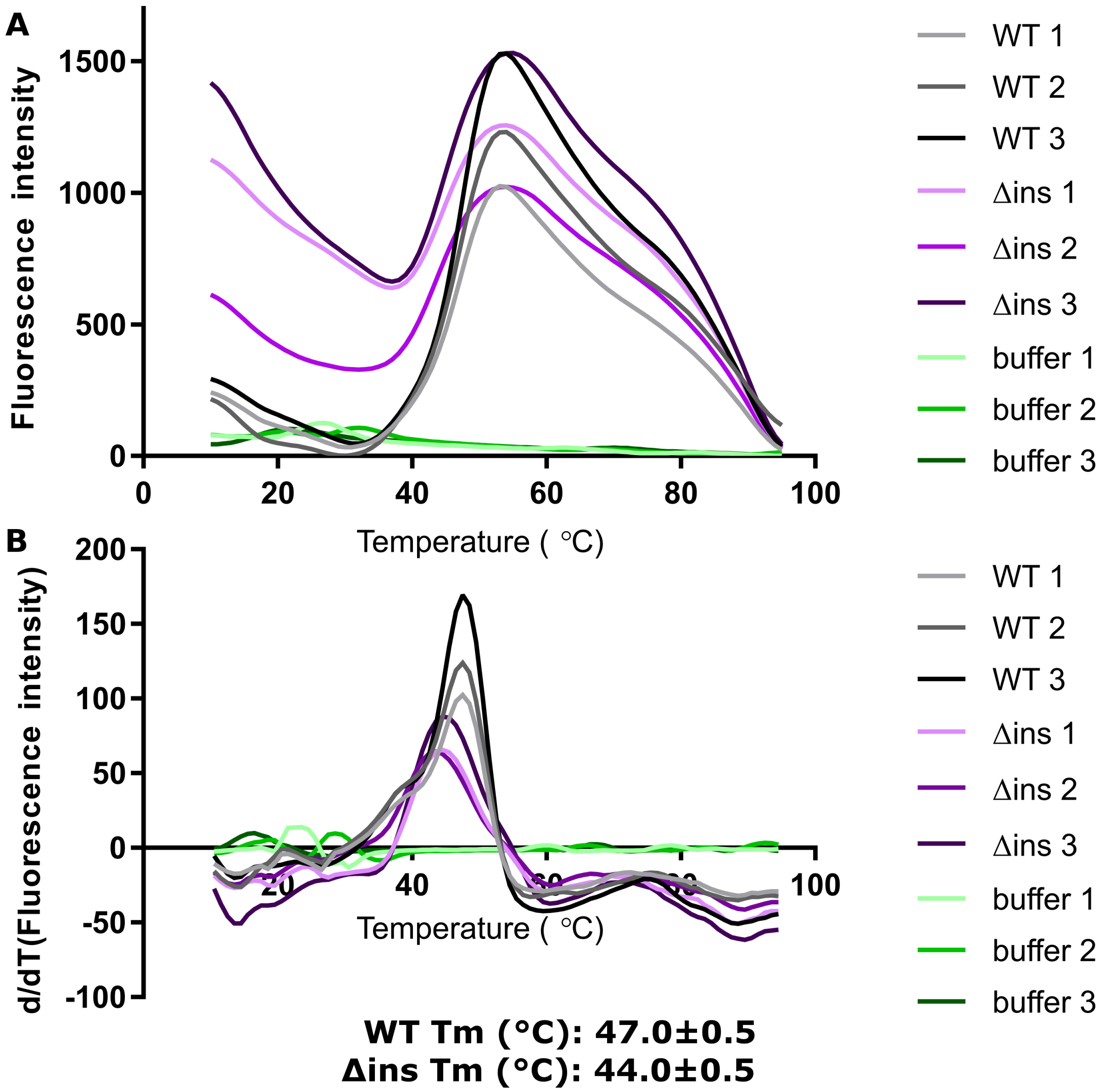

Figure S5:

ThermoFluor assay data for wild type kUNG and kUNGΔins. 25 µL reactions included 5× Sypro Orange (Life Technologies) and protein at a concentration of 1 mg/mL in kUNG purification buffer B (25 mM Tris-HCl, pH 7.5, 200 mM NaCl, 1mM EDTA, 1% glycerol). Temperature was increased from 10° C to 95° C in 0.5° C increments with a dwell time of 10 s at each temperature. Increase in fluorescence was monitored using a Bio-Rad MyiQ real-time PCR detection system. **A**: Raw data of triplicate experiments for wild type kUNG (grey/black), kUNGΔins (magenta), and kUNG purification buffer B (green). Baselines were corrected individually and curves were normalised according to maximum total fluorescence. **B**: First derivative curve of thermal unfolding data. T_m values were determined as the maxima of first derivative curves.

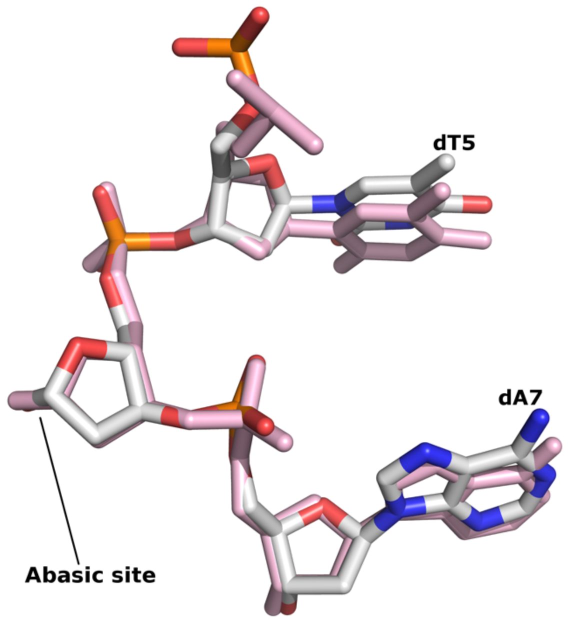

Figure S6:

Comparison of DNA backbone conformations at the abasic position left after uracil cleavage in kUNG and hUNG (PDB entry 2SSP (19)) with these structures aligned on protein chains. DNA from the kUNG-dsDNA structure is shown as sticks with grey carbons, DNA from 2SSP is shown as pink sticks. Despite extensive differences in the DNA conformations seen elsewhere in these structures, the positon of the backbone around the abasic site is strikingly similar allowing canonical catalysis to take place.

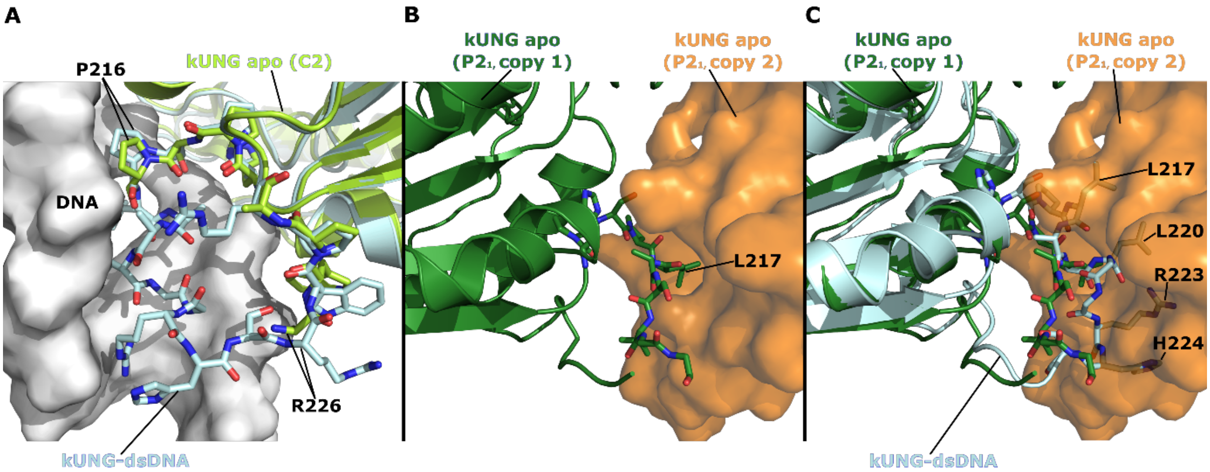

Figure S7:

Leucine loop regions of unbound kUNG structures. **A**: Comparison of the unbound kUNG structure solved in space group C2 to the kUNG-dsDNA structure. kUNG from the kUNG-dsDNA structure is shown in pale cyan, DNA is shown as a grey surface. Leucine loop residues present in the electron density (light green sticks) take up similar positions to the equivalent residues in the kUNG-dsDNA structure. Residues L217 to S225 were not ordered in the crystal. **B**: The leucine loop region of the unbound kUNG structure solved in space group $P2_1$. Two symmetry-related copies of kUNG are shown, in green and orange, respectively. L217 of copy 1 contacts a hydrophobic pocket in copy 2. **C**: The same as in **B** with kUNG from the kUNG-dsDNA structure overlaid in pale cyan. The position of the leucine loop in copy 1 the unbound kUNG structure is precluded from taking up the same position as in the kUNG-dsDNA structure by steric hindrance from copy 2. Key residues in the kUNG-dsDNA structure which would clash with copy 2 of the unbound kUNG structure are labelled

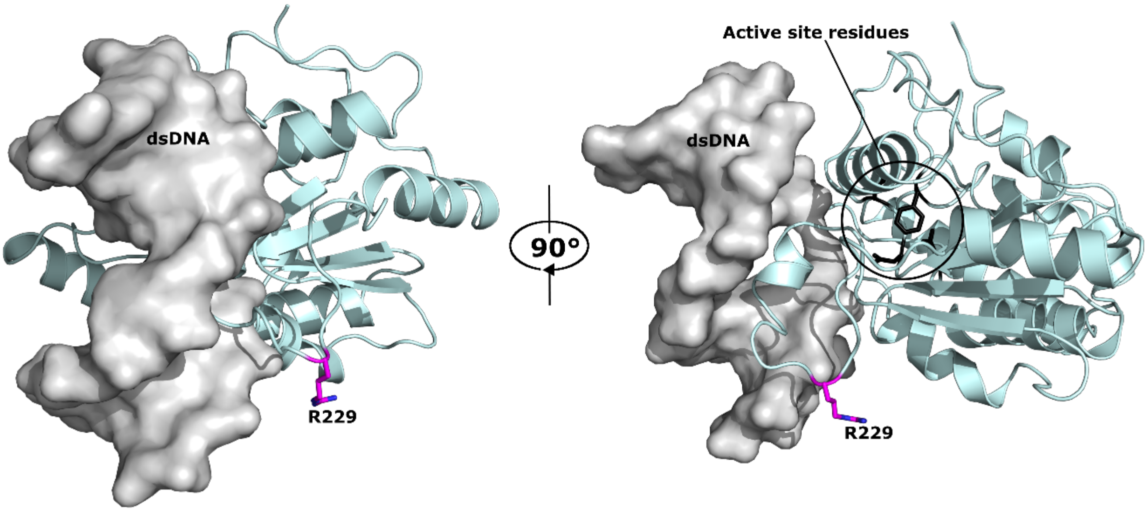

Figure S8:

R226 in the kUNG-dsDNA complex. The side chain of R229 (magenta) is solvent exposed, making no contacts with DNA. R229 is also distal from the kUNG active site, active site residues D91, Y93, F104, and N149 are shown in black.

5NNU: kUNG-dsDNA

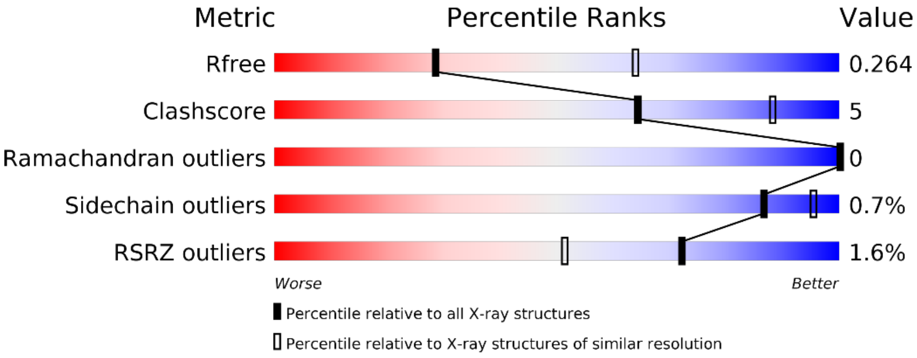

5NNH: kUNG apo form, space group: C2

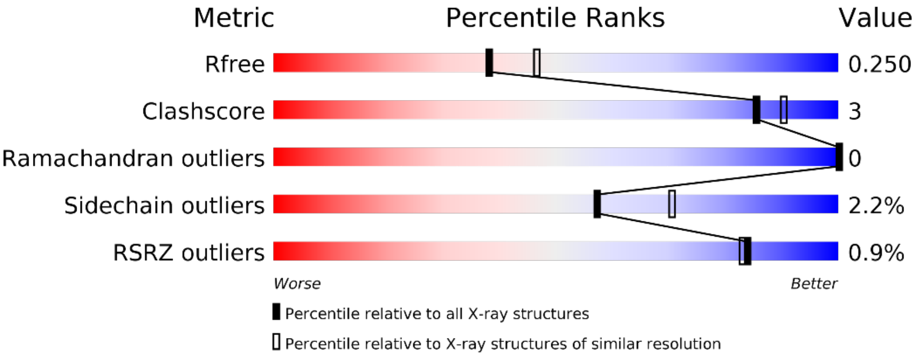

5NN7: kUNG apo form, space group : $P2_1$

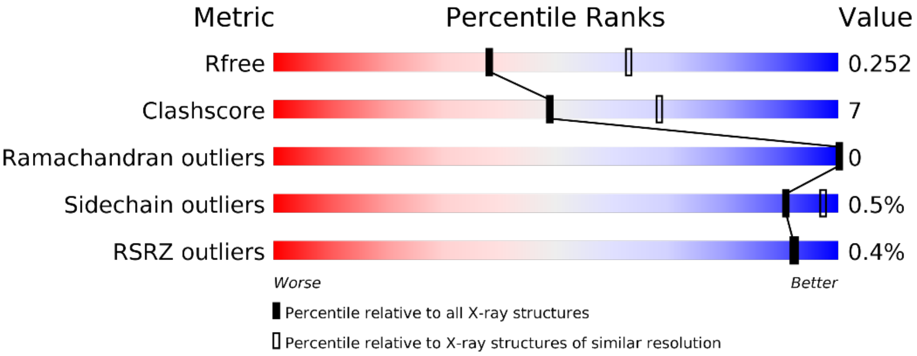

Figure S9:

Percentile scores for global validation metrics of the kUNG-dsDNA structure and apo form kUNG structures. Graphics are taken from validation reports provided by PDBe upon submission of the structures.
